# A Repeated and Delayed Homologous Challenge Study Evaluating the Durability of Protection Induced by the Live Attenuated ASF Vaccine Candidate ASFV-G-ΔI177L/ΔLVR

**DOI:** 10.3390/vaccines14070561

**Published:** 2026-06-25

**Authors:** Xinghua Zheng, Yeonji Kim, Sun A. Choi, Su Jin Lee, Seung Pyo Shin, Se Young Lee, Wonjun Kim, Seong Cheol Moon, Yongwoo Shin, Do Soon Kim, Byung-chul Shin, Sua Choi, Ji-yun Sung, Garam Kim, Weonhwa Jheong, Jung Hyang Sur

**Affiliations:** 1 Central Research & Development Institute, Komipharm International Co., Ltd., Siheung-si 15094, Republic of Korea; xinghua@komipharm.com (X.Z.); dktjschl@komipharm.com (S.A.C.); leesoot@komipharm.com (S.J.L.); sinseng1234@komipharm.com (S.P.S.); man32@komipharm.com (S.Y.L.); moonvet@komipharm.com (S.C.M.); kimds202345@komipharm.com (D.S.K.); 2 Wildlife Disease Response Team, National Institute of Wildlife Disease Control and Prevention (NIWDC), Ministry of Climate, Energy and Environment (MCEE), Songamgil 1, Gwangsan-gu, Gwangju 62407, Republic of Korea; yeonchi16@korea.kr (Y.K.); kwj22081@korea.kr (W.K.); jams80@korea.kr (Y.S.); njchul2@korea.kr (B.-c.S.); choisua@korea.kr (S.C.); jo8216@korea.kr (J.-y.S.); garam1204@korea.kr (G.K.)

**Keywords:** African Swine Fever Virus (ASFV), live attenuated vaccine, ASFV-G-ΔI177L/ΔLVR, repeated exposure, protective efficacy, endemic control

## Abstract

Background/Objectives: African swine fever (ASF) is a highly lethal disease of domestic pigs and wild suids that continues to cause substantial economic losses worldwide. Despite recent progress in live attenuated ASF vaccine development, evidence supporting durable protection under repeated exposure conditions representative of endemic settings remains limited. Here, we assessed the long-term safety and protective efficacy of a live attenuated ASFV-G-ΔI177L/ΔLVR vaccine using a repeated-challenge experimental design intended to model re-exposure in ASF-endemic regions. Methods: Vaccinated pigs were subjected to homologous virulent ASF virus challenges at multiple intervals, including repeated challenges (three sequential inoculations) and single challenges administered at 8 and 12 weeks post-vaccination. Results: Across all challenge regimens, vaccinated animals survived and remained clinically healthy, including those receiving three challenges, supporting sustained protection under repeated exposure pressure. Animals challenged at 8 or 12 weeks post-vaccination likewise exhibited complete survival, indicating maintained efficacy through at least 12 weeks. No vaccine-associated adverse clinical outcomes were detected over the study period, and post-challenge viral shedding was minimal. Conclusions: Overall, these data demonstrate that the candidate live attenuated ASF vaccine provides excellent protective efficacy and confers sustained protection against homologous ASF virus infection. This result is expected to be equally applicable under repeated exposure conditions in regions with unstable ASF biosecurity, making it a sufficiently promising model experiment for field application in ASF epidemic areas. However, this is still a vaccine variant, and further studies are planned to evaluate its genomic stability and transmissibility.

## 1. Introduction

African swine fever (ASF) is a highly lethal and often fatal hemorrhagic disease affecting domestic pigs and wild boars, caused by the African swine fever virus (ASFV). ASFV is a large double-stranded DNA virus belonging to the family *Asfarviridae* [1]. First identified in Kenya in the 1920s, ASF has since spread globally, causing significant economic losses to the swine industry due to high mortality rates, trade restrictions, and population reduction measures [2,3]. In endemic regions such as sub-Saharan Africa, parts of Europe, and Asia, the virus persists through complex transmission cycles involving wild suids, ticks (*Ornithodoros* spp.), and domestic pigs, making control difficult without effective vaccination [4,5,6,7]. Although existing control strategies, including biosecurity measures and culling, have proven insufficient to prevent outbreaks, particularly in areas of persistent virus circulation, ASF control is further complicated in certain regions by the prevalence of backyard farms [8,9,10]. The development of a safe and effective vaccine has emerged as a top priority, and live attenuated vaccines (LAVs) have emerged as promising candidates due to their ability to induce robust cellular and humoral immune responses mimicking natural infection [5,11].

Among the recently developed LAV strains, ASFV-G-ΔI177L represented an important milestone in ASF vaccine development. Developed through deletion of the I177L gene (MGF505-7R), ASFV-G-ΔI177L demonstrated strong protective efficacy against a homologous challenge and showed initial promise as a vaccine candidate [12,13,14]. However, this strain was restricted to replication in primary porcine alveolar macrophages, severely limiting commercial-scale production feasibility. Moreover, recent studies have revealed concerning genetic instability of ASFV-G-ΔI177L, with documented reversion to virulence during both experimental studies and vaccination trials [15]. Additional safety concerns include vaccine virus shedding through boar semen, raising risks for reproductive transmission [16]. Concerns regarding genetic stability have also been documented under field conditions, as a recent study in Vietnam reported the detection of a virulent ASFV-G-ΔI177L-derived variant in an unvaccinated pig farm [17]. Overall, these findings emphasize the need to improve attenuation strategies for ASFV live attenuated vaccines, including the incorporation of multiple genomic deletions to enhance vaccine safety and genetic stability.

To overcome commercial production limitations while maintaining vaccine efficacy and safety, ASFV-G-ΔI177L was adapted for efficient propagation in a stable Plum Island porcine epithelial cell (PIPEC) through serial passaging, resulting in the development of the derivative strain ASFV-G-ΔI177L/ΔLVR [18]. This new strain retained equivalent levels of attenuation, immunogenicity, and protective efficacy compared to the parent ASFV-G-ΔI177L while enabling scalable vaccine production in established cell culture systems. The adaptation process involved a naturally occurring 10,842 bp deletion in the Left Variable Region (LVR), removing several MGF360 and MGF300 family genes as well as the *X69R* gene [18].

The scientific rationale for the LVR deletion stems from well-documented observations of ASFV adaptation to established cell lines. LVR deletions are commonly observed during ASFV adaptation to cell culture and are known to affect components of Multi-Gene Families (MGFs), particularly MGF505 and MGF360 [19]. This genomic change was not artificially introduced but rather represents an essential adaptation that enables efficient viral replication in the PIPEC environment. The LVR deletion likely contributes to altered cell tropism by removing genes that restrict growth outside primary macrophages, thereby facilitating commercial-scale production while maintaining the vaccine’s inherent safety and protective characteristics. This natural adaptation addresses the critical production bottleneck that limited the practical application of the parent strain, positioning ASFV-G-ΔI177L/ΔLVR as a more viable candidate for commercial vaccine development [18].

These LAVs have demonstrated high efficacy in preventing clinical disease following challenge infections with wild-type homologous viruses as well as field isolates currently circulating in Southeast and Northeast Asian regions [13,20,21]. However, gaps remain in understanding long-term efficacy, particularly under conditions simulating endemic exposure that include repeated viral infections reflecting the real-world scenario of multiple infections over time. Although the immunological correlates of protection against these attenuated vaccine strains are not fully understood, studies correlating increased peripheral blood mononuclear cell counts, interferon (IFN)γ-producing cell numbers, and various interleukins (ILs) with strong persistent vaccine antibody levels following ASF attenuated live vaccine administration have been reported [22,23,24].

Therefore, this study utilized a comprehensive experimental design incorporating repeated homologous virus challenge experiments and delayed challenge protocols at 8 and 12 weeks post-vaccination to evaluate both vaccine efficacy and the persistence of ASF protective antibodies in pigs. Furthermore, this clinical trial included serum cytokine analysis to predict correlations with immune responses, albeit with limited experimental scope, thereby providing a comprehensive assessment of live attenuated ASF vaccine performance. One finding was the sustained detection of vaccine genome in blood and maintenance of high vaccine antibody concentrations in most individuals after a single vaccination. However, some individuals maintained high levels of vaccine antibodies for 4 months despite low levels or complete disappearance of the vaccine genome in their blood. Therefore, to understand the correlation between this phenomenon and cellular immune markers, we compared and analyzed the associated IFN-γ, IL-12/IL-23 p40, and the anti-inflammatory cytokine marker interleukin-1 receptor antagonist (IL-1ra).

The experimental design included repeated challenge infections up to three doses following vaccine antibody formation, along with a delayed challenge protocol to assess sustained immunogenicity post-vaccination. This protocol was designed to evaluate durability against cumulative viral pressure under conditions mimicking epidemic conditions. Results demonstrated complete protection of all tested animals and the absence of clinical disease in all groups, confirming the vaccine’s safety and sustained protective efficacy. Although all vaccinated animals survived the challenge infection, the small sample size limits statistical inference. These findings, building on previous studies demonstrating initial protective effects [14,18,25,26], extend this protection to long-term and multiple exposure scenarios, laying the groundwork for field trials in ASF-endemic regions.

Consequently, this type of clinical trial is considered a viable field clinical trial model. It is expected to provide valuable data for strategically evaluating vaccine policy efficacy, particularly in specific Asian regions where ASF occurs continuously and periodically, and where numerous backyard farms of varying sizes exist.

## 2. Materials and Methods

### 2.1. Cell Culture and Virus

The preparation and characterization of the live attenuated ASFV vaccine candidate ASFV-G-ΔI177L/ΔLVR were conducted as previously described in detail [15], in accordance with World Organization for Animal Health (WOAH) guidelines. Briefly, the vaccine strain was derived from an eighth-passage (P8) ASF-LAV provided by the USDA and further passaged in Plum Island porcine epithelial cells (PIPECs) stably expressing bovine αVβ6 integrin. Cells were maintained under standard culture conditions, and viral replication was monitored by mCherry fluorescence [18]. Following serial passaging, the P19 virus stock exhibiting the highest titer and confirmed genetic stability was selected as the master seed virus.

Virus titration was performed using primary swine macrophage cultures, with infectious titers determined by hemadsorption assay and calculated using the Reed and Muench method [27]. Domestic pigs were immunized with the ASFV-G-ΔI177L/ΔLVR vaccine and subsequently challenged with a homologous, highly virulent field strain, Korea/wildboar/ASFV-Hwacheon/2020 (GenBank accession no. OR159219.1), supplied by the Ministry of Environment of the Republic of Korea. Experimental challenge regimens included repeated and delayed homologous inoculations to evaluate long-term safety and durability of protection.

### 2.2. Animal Experiments

Animal experiments were conducted under Animal Biosafety Level 3 (ABSL-3) conditions at the Wildlife Disease Response Team, National Institute of Wildlife Disease Control and Prevention (NIWDC), Ministry of Environment. All experimental protocols were approved by the Institutional Animal Care and Use Committee (Protocol NIWDC-IACUC-2025-002). The experimental animals were 8-week-old commercial breed pigs. Upon arrival at the facility, they underwent a minimum one-week acclimation period and were observed to confirm the absence of major swine diseases that could affect the experiment. Blood samples were collected during the acclimation period to confirm infection status for Porcine Circovirus 2, Mycoplasma hyopneumoniae, and Porcine Reproductive and Respiratory Syndrome Virus before use. The animals were divided into three distinct trial models (T-1, T-2, and T-3) for intramuscular vaccination, and a challenge control (C-1, C-2, and C-3) group was assigned to each model. Four pigs were assigned to each trial model for challenge vaccination, and two pigs were assigned to each challenge control group within each model. In brief, all animals received the same vaccine dose but were challenged at different time points. The T-1 group received repeated challenge infections at 4, 8, and 12 weeks; the T-2 group received a single challenge infection at 8 weeks, and the T-3 group received a single challenge infection at 12 weeks (Table 1). While this study acknowledges the small sample size, it presents the findings as preliminary evidence to support further research rather than as definitive proof of the candidate vaccine’s efficacy. Furthermore, as a preliminary study requiring validation according to current international standards, it aims to provide proof-of-concept data to support the feasibility of large-scale confirmatory clinical trials that comply with WOAH standards [10].

The experimental groups, divided into three trial models, were intramuscularly inoculated with 1 mL of ASFV-G-∆I177L/∆LVR at 10^3^ TCID_50_ on the same day. The challenge virus, a highly pathogenic Korean field strain of ASFV, Hwacheon/2020, was prepared at 10^2^ HAD_50_ for intramuscular inoculation in each experimental model. Following vaccination, clinical signs and rectal temperature were measured daily after challenge for each trial model. All samples were used as reference materials for analyzing vaccine antibodies, vaccine genome, and serum cytokines per ASF challenge interval. Animals were euthanized and necropsied at the end of the experimental period for each model.

(i) T-1 Group (V1–V4): Four healthy 8-week-old pigs were used as experimental animals. Whole blood, oral, and rectal swab samples were collected at 0, 3, 7, 10, 14, 17, 21, 24, and 28 days post-vaccination (dpv). These samples were used for vaccine genome copy number, antibody, and cytokine analysis. The primary challenge was conducted on day 28 post-vaccination. Samples were collected for the same purposes at 3, 7, 10, 14, 17, 21, 24, and 28 days post-challenge (dpc). The second challenge was performed 28 days after the first challenge. Samples were collected at 4, 7, 11, 14, 18, 21, and 24 dpc following the second challenge. The final third challenge was conducted on day 24 after the second challenge. Following the third challenge, samples were collected on days 3, 7, 11, 14, 18, and 21, after which the animals were euthanized. The euthanized animals were then necropsied for pathological analysis.
(ii) T-2 Group (V5–V8): Four healthy 8-week-old pigs were used as test animals. Whole blood, oral, and rectal swab samples were collected at the same time points as the T-1 group after vaccination. The challenge was performed 59 dpv, and clinical symptoms were monitored until day 101. All samples were analyzed using the same procedures as those applied to the T-1 group.
(iii) T-3 Group (V9–V12): Four healthy 8-week-old pigs were used as test animals. Whole blood, oral, and rectal swab samples were collected at the same time points as the T-1 group after vaccination. Challenge was performed at 87 dpv, and clinical symptoms were observed until day 101. All samples were analyzed using the same procedures as those applied to the T-1 group.
(iv) Positive control: Three positive control groups (C–13~C14, C–15~C16, and C–17~C18) were assigned two challenge controls per trial model. Challenge inoculation was performed on the control group at the same time as the challenge inoculation for each trial model, and they were used as positive controls. Furthermore, sample collection was also performed at the same time for each trial model, collecting all samples until the time of death and performing necropsy.

### 2.3. Quantitative Real Time PCR (qPCR) for Detection of the ASFV Genome

qPCR was used to detect ASFV DNA in whole blood, oral swab, and rectal swab samples collected before and after vaccination, as well as after the ASFV challenge, in each experimental model. The experimental procedures were conducted according to previously described methods [15,24], with minor modifications. Briefly, whole blood, oral swab, and rectal swab samples were collected at each vaccination and post-challenge time point. ASFV DNA was extracted using the RSC Whole Blood DNA Kit and RSC Tissue DNA Kit (Maxwell^®^; Promega, Madison, WI, USA) with the Maxwell^®^ CSC 48 Instrument IVD (Promega), following the manufacturer’s instructions. The DNA extraction procedure was identical for all sample types.

For each sample, 300 µL of whole blood or swab suspension was transferred to a reaction tube, followed by the addition of 20 µL of proteinase K and 200 µL of lysis buffer. Samples were vortexed for 10 s and incubated at 56 °C for 20 min using a heating block. DNA purification was subsequently performed according to the manufacturer’s protocol, and nucleic acids were eluted in 50 µL of elution buffer.

ASFV-specific qPCR was performed using the VetMAX™ African Swine Fever Virus Detection Kit (Thermo Fisher Scientific, Waltham, MA, USA), which is validated and certified by the WOAH for ASFV detection targeting the p72 gene [25]. Samples with a cycle threshold (Ct) value < 45 were considered positive.

### 2.4. ASFV Antibody Detection

Whole blood samples were collected from animals vaccinated with ASFV-G-∆I177L/∆LVR and subsequently challenged. Serum was separated by centrifugation and stored until analysis. ASFV-specific antibody levels in serum were determined using the ID Screen^®^ African Swine Fever Virus competitive enzyme-linked immunosorbent assay (cELISA; IDvet, Grabels, France), according to the manufacturer’s instructions.

The sample-to-negative control ratio (S/N%) was calculated using the formula provided by the manufacturer. Serum samples with an S/N% ≤ 40% were considered positive for ASFV-specific antibodies. Samples with an S/N% ≥ 50% were classified as suspected and were reanalyzed to confirm the results.

### 2.5. Serum Cytokine Analysis

Porcine cytokine concentrations were determined using DuoSet™ ELISA Development Systems for Porcine IFN-γ (Cat#: DY985), Porcine IL-1ra/IL-1F3 (Cat#: DY780), and Porcine IL-12/IL-23 p40 (Cat#: DY912) (R&D Systems, Minneapolis, MN, USA) according to the manufacturers’instructions. High-binding 96-well microplates (SPL Life Sciences, SPL38096, Pocheon-si, Gyeonggi-do, Republic of Korea, SPL38096) were coated with capture antibody overnight at room temperature (RT), washed, and blocked with 1% bovine serum albumin in phosphate-buffered saline for 1 h at RT. Standards and samples were then added, followed by sequential incubation with biotinylated detection antibody and streptavidin–horseradish peroxidase. Color was developed using 1-Step™ Ultra TMB-ELISA Substrate (Thermo Scientific, 34028, Waltham, MA, USA), and the reaction was stopped with stop solution (Invitrogen, SS04, Waltham, MA, USA). Absorbance was measured at 450 nm with reference correction at 540 nm using an Epoch 2 microplate reader (BioTek, Santa Clara, CA, USA), and corrected values (OD450–OD540) were used for analysis. Cytokine concentrations were calculated from standard curves generated using serially diluted standards for each assay.

## 3. Results

### 3.1. Protective Efficacy of ASFV-G-ΔI177L/ΔLVR Vaccination Against Multiple and Delayed Challenges in an Experimental Model

**Figure 1:**
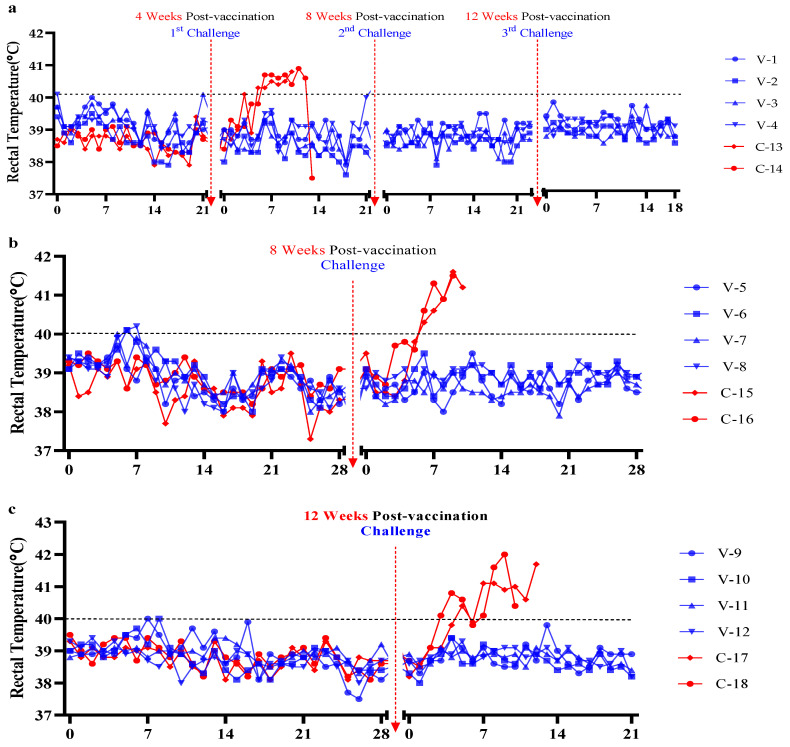
Kinetics of rectal temperature values in pigs intramuscular inoculated with ASFV-G-ΔI177L/ΔLVR/10^3^TCID_50_ and after the challenge with ASFV-Hwacheon/2020 (G-II)/10^2^HAD_50_. The dotted line indicates the threshold for fever at 40 °C. Each curve represents an individual animal’s values in each group. Data from control animals are depicted in red. (**a**) Triple challenge; (**b**) Challenge at Week 8; (**c**) Challenge at Week 12.

The trial group was divided into three subgroups. The first group (T-1) underwent three repeated challenge inoculations using the highly virulent field ASFV strain Hwacheon/2020 to evaluate the durability of protective immunity against ASFV-G-ΔI177L/ΔLVR. The other groups (T-2 and T-3) were challenged with 10^2^ HAD virulent field ASFV-Hwacheon/2020 at planned time points for each experimental model to confirm the protective capacity of the ASFV-G-ΔI177L/ΔLVR vaccine at 8 and 12 weeks post-vaccination. Simultaneously, two positive control animals per group were challenged with virulent field ASFV-Hwacheon/2020 at the challenge time points for each group and evaluated in comparison to the vaccine groups. All six animals in the positive control group, which received only the virulent field ASFV-Hwacheon/2020, exhibited a body temperature rise above 40 °C by days 5–6 post-infection (pi) and showed disease-related clinical symptoms including anorexia, depression, cyanosis, staggering gait, and diarrhea (Figure 1a). Symptoms progressively worsened over time, and by days 8–12 pi, the animals either died or were euthanized due to extreme distress. In contrast, animals challenged after vaccination in all three distinct trial models maintained healthy status until market age without a fever response or specific clinical findings. Detailed results by inoculation model are as follows.

T-1 Group (V1–V4): Four 8-week-old pigs were initially vaccinated with the ASFV-G-ΔI177L/ΔLVR vaccine. Transient febrile responses at 40 °C were observed in two animals but resolved spontaneously, with no specific clinical symptoms detected through day 28 post-vaccination. On day 28 post-vaccination, the first challenge was administered intramuscularly (IM) with 1 mL of 10^2^ HAD virulent field ASFV-Hwacheon/2020. No specific febrile responses were observed during the 28-day observation period, and all animals remained clinically normal without any ASF-related symptoms prior to the second challenge. The second challenge was conducted on day 28 post-first challenge, with no specific clinical findings including febrile responses observed during the monitoring period. The final third challenge was performed on day 28 post-second challenge and monitored for 18 days, during which all animals demonstrated normal vigorous feeding behavior and maintained healthy conditions. Upon necropsy of all four animals following three sequential challenges, thorough examination of major organs revealed no detectable ASF lesions in most organs. However, mild congestion and hemorrhage were observed in the gastro-hepatic lymph nodes of all four animals.

Positive Control Group (C-13, C-14): Two pigs served as positive controls and were inoculated IM with 1 mL of 10^2^ HAD virulent field ASFV-Hwacheon/2020 using the same method at the time of the first challenge. High fever exceeding 40 °C persisted for 6–9 days beginning on day 4 post-challenge (Figure 1a). One animal died on day 10 and the other on day 13 post-challenge. Necropsy examination revealed severe enlargement and congestion/hemorrhage in most major organs, including submandibular lymph nodes and gastro-hepatic lymph nodes, along with splenomegaly and petechial hemorrhages in the kidneys, representing typical ASF necropsy findings.

T-2 Group (V5–V8): Four 8-week-old pigs were initially vaccinated with ASFV-G-ΔI177L/ΔLVR vaccine. Transient fever was observed in two animals through day 28 post-vaccination, but no specific clinical symptoms were detected (Figure 1b). After 59 days post-vaccination, the first challenge was administered IM with 1 mL of 10^2^ HAD virulent field ASFV-Hwacheon/2020. During the approximately 46-day observation period, the animals showed no ASF-related symptoms and maintained clinically normal health status. All four animals in the group were euthanized on day 46 for necropsy examination. Thorough examination of major organs revealed no detectable ASF lesions in most organs. However, similar to the T-1 group, mild congestion and hemorrhage were observed in the gastro-hepatic lymph nodes.

Positive Control Group (C-15, C-16): Two pigs served as positive controls and were inoculated IM with 1 mL of 10^2^ HAD virulent field ASFV-Hwacheon/2020 using the same method at the time of the T-2 group’s first challenge. High fever exceeding 40 °C persisted for 5–10 days beginning on day 4 post-challenge (Figure 1b). One animal died on day 9 and the other on day 10 post-challenge. Necropsy examination following death revealed typical ASF necropsy findings in most major organs.

T-3 Group (V9–V12): Four 8-week-old pigs were initially vaccinated with ASFV-G-ΔI177L/ΔLVR vaccine. Transient fever was observed through day 28 post-vaccination, but no specific clinical symptoms were detected (Figure 1c). After 87 days post-vaccination, the first challenge was administered IM with 1 mL of 10^2^ HAD virulent field ASFV-Hwacheon/2020. During the 18-day observation period, the animals showed no ASF-related symptoms and maintained clinically normal health status. Therefore, the immunogenicity of the ASF vaccine persisted until a very late time point, approximately 3 months post-vaccination, demonstrating protective efficacy even against highly pathogenic field-isolated ASFV. All four animals in this group were also euthanized on day 18 post-challenge for necropsy examination. Thorough examination of major organs revealed no detectable ASF lesions in most organs. However, similar to the T-1 and T-2 groups, mild congestion and hemorrhage were observed in the gastro-hepatic lymph nodes.

Positive Control Group (C-17, C-18): Two pigs served as positive controls and were inoculated IM with 1 mL of 10^2^ HAD virulent field ASFV-Hwacheon/2020 using the same method at the time of the T-3 group’s first challenge. High fever exceeding 40 °C persisted for 5–10 days beginning on day 4 post-challenge (Figure 1c). One animal died on day 10 and the other on day 12 post-challenge. Necropsy examination following death revealed typical ASF necropsy findings in most major organs.

### 3.2. Evaluation of ASF Vaccine Antibodies in Challenged Pigs After ASFV-G-ΔI177L/ΔLVR Vaccination

**Figure 2:**
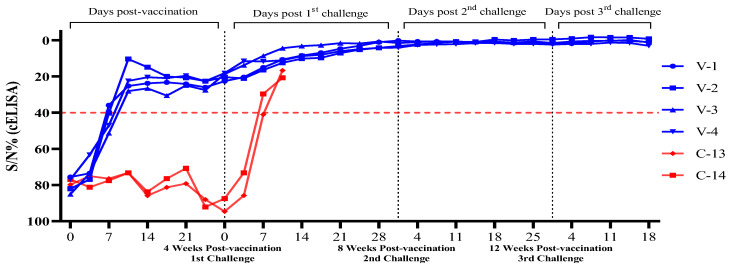
This graph shows vaccine antibody titers measured by competitive enzyme-linked immunosorbent assay (cELISA) at all intervals following three challenges administered at 4-week intervals after vaccination. Antibodies meeting the positive threshold (S/N% ≤ 40) were detected starting on day 7 post-vaccination, and higher vaccine antibody levels were maintained with each subsequent challenge.

**Figure 3:**
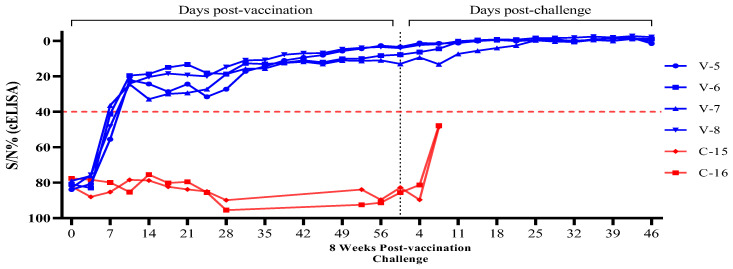
This graph shows challenge results at 8 weeks post-vaccination and vaccine antibody levels measured by competitive enzyme-linked immunosorbent assay (cELISA). Antibodies meeting the positive threshold (S/N% ≤ 40) were detected starting 7 days post-vaccination. Higher vaccine antibody levels were maintained over time, and even higher antibody levels were confirmed at 8 weeks post-challenge.

**Figure 4:**
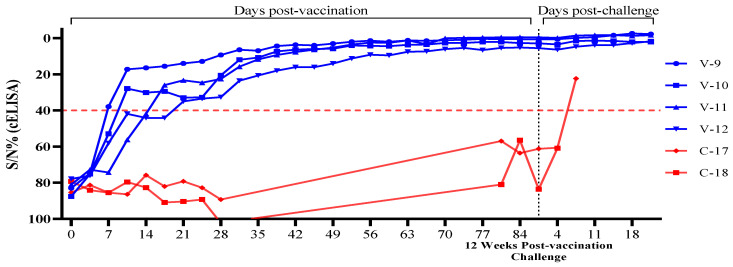
This graph shows challenge results at 12 weeks post-vaccination and measured vaccine antibody titers via competitive enzyme-linked immunosorbent assay (cELISA). Antibody positivity (S/N% ≤ 40) was confirmed starting 7 days post-vaccination. Higher vaccine antibodies were maintained over time, and even higher antibody titers were confirmed at 12 weeks post-challenge.

Antibody detection using cELISA was interpreted as positive when S/N% values were <40%. Whole blood samples were collected from each group at planned time points post-vaccination to evaluate antibody responses to the vaccine (Figure 2, Figure 3 and Figure 4 and Table 2, Table 3 and Table 4).

T-1 Group (V1–V4): In the ASFV-G-∆I177L/∆LVR vaccinated group, antibody positive responses were detected in one out of four animals on day 7 post-vaccination (25%, 1/4). From day 10 onwards, moderate levels of positive antibody responses (S/N% = 21.48 ± 6.75) were confirmed in all vaccinated animals. At 28 dpv, relatively high levels of positive antibody responses (S/N% = 19.88 ± 1.72) persisted. Following the first challenge, higher antibody levels (S/N% = 9.58 ± 3.12) were maintained. Antibody levels at the time points of the second and third challenges showed progressively higher concentrations that were sustained until euthanasia. In contrast, the ASF positive control group (C13–C14) received no vaccination and was challenged simultaneously with the T-1 group’s first challenge at 28 dpv. Therefore, antibodies against field-isolated ASFV began to be detected in the control group from day 7 post-challenge, but all animals died by day 14 (Figure 2 and Table 2).

T-2 Group (V5–V8): Positive antibody responses were detected in one out of four animals on day 7 post-vaccination (25%, 1/4). From day 10 onwards, all vaccinated animals showed seroconversion to moderate positive antibody responses, with vaccine antibody levels dramatically increasing over time to reach S/N% = 6.98 ± 3.81 at the challenge time point (day 59). Following the challenge on day 59, even higher levels of ASF antibodies (S/N% = 2.28 ± 2.92) were observed (Figure 3 and Table 3).

T-3 Group (V9–V12): Positive antibodies were detected in one out of four animals on day 7 post-vaccination, with only three out of four animals showing positive responses by day 17 dpv. Notably, animal V-12 in this group showed a relatively delayed seroconversion, testing positive only on day 21 dpv. Consequently, vaccine antibody levels remained at high levels over time, with strong ASF protective antibodies (S/N% = 2.60 ± 2.45) persisting at the challenge time point (day 87). Following challenge on day 87, even higher levels of ASF antibodies (S/N% = 1.13 ± 2.25) were confirmed. Notably, the T-3 group demonstrated sustained ASF antibody levels above initial levels without apparent antibody half-life decay, maintaining vaccine antibody persistence until challenge even at 3 months post-vaccination (Figure 4 and Table 4).

### 3.3. Evaluation of Viremia Against Multiple and Delayed Challenges Following ASFV-G-ΔI177L/ΔLVR Vaccination

#### Vaccine-Associated Viremia Assessment

Vaccine-associated viremia following ASFV-G-∆I177L/∆LVR vaccination was assessed using whole blood, serum, and oral and rectal swabs according to the planned protocol. Evaluation of vaccine genome (ASFV p72 gene) was performed based on Ct values obtained from real-time PCR reactions targeting the ASF gene. A comparative analysis between whole blood and serum was conducted throughout the entire experimental period. For detailed whole blood Ct values by trial group, refer to the Supplementary Tables. Serum, oral and rectal Ct values for each trial group are summarized in both graphical and tabular formats in the Supplementary Figures and Tables.

**Figure 5:**
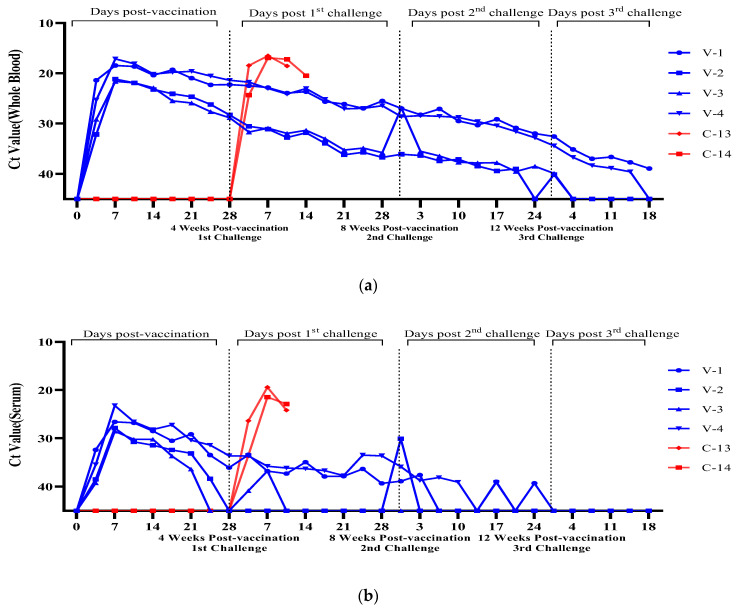
(**a**) This graph shows vaccine-associated viremia in whole blood after three repeated challenges at 4 weeks post-vaccination. The positive detection criterion was cycle threshold (Ct) ≤ 45. With each subsequent challenge, vaccine-associated viremia was confirmed to be negative in all three animals except one at the time of euthanasia. (**b**) This graph shows vaccine-associated viremia observed in serum samples taken at intervals following three repeated challenges 4 weeks after vaccination. The positive detection criterion was Ct ≤ 45. Although the challenge was repeated three times, vaccine-associated viremia was confirmed to be at undetectable levels in all animals starting 8 weeks prior to euthanasia.

T-1 Group (V1–V4): On day 28 post-vaccination dpv, the whole blood Ct value was measured at an average of 25.18 ± 3.39. Following the first challenge at 28 dpv, the Ct value at 31 dpc was 29.64 ± 3.79, while the Ct value at the end of the second challenge was 36.75 ± 3.33. Subsequently, after the third challenge (at euthanasia), three out of four animals tested negative for the ASF target gene, with only one animal testing positive. The mean Ct value was 43.48 ± 2.63, indicating very low levels of viremia (Figure 5a and Table S1). Comparing vaccine-associated viremia in serum, the Ct value at the end of the first challenge was 37.45 ± 5.38, while the ASF target gene was undetectable at the end of both the second and third challenges. These results demonstrated that whole blood provides more accurate viremia detection than serum for post-vaccination assessment (Figure 5b and Table S2). Regarding oral swab analysis, vaccine-associated viremia (virus shedding) was confirmed at considerably lower levels (44.32 ± 1.92) compared to whole blood Ct values in most animals after the first challenge, with no virus shedding detected after the third challenge (Figure S1 and Table S3). Rectal swabs showed significantly low levels after the first challenge, but no virus shedding was detected from the second challenge onwards (Figure S2 and Table S4).

**Figure 6:**
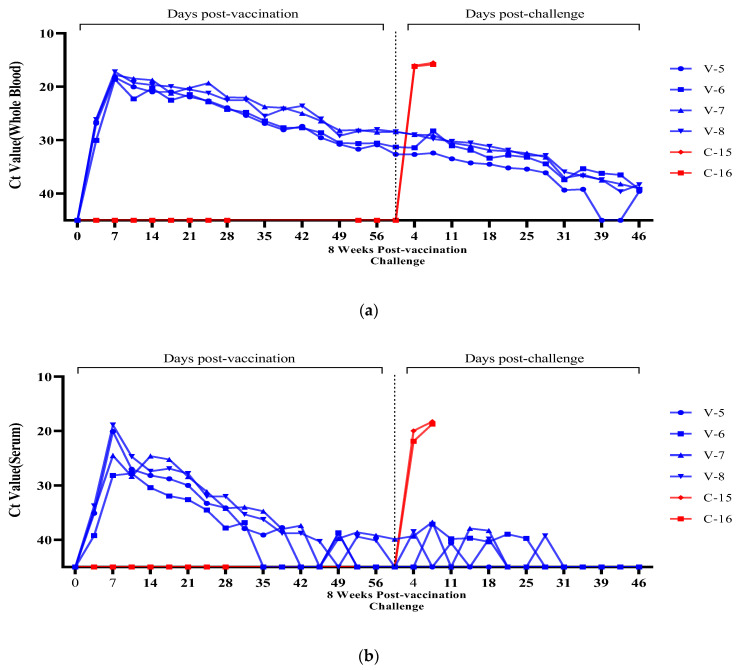
(**a**) This graph shows vaccine-associated viremia in whole blood following an 8-week challenge after vaccination. The positivity threshold is Ct ≤ 45. A sharp decrease in vaccine-associated viremia is observed over time. (**b**) This graph shows vaccine-associated viremia observed in serum samples taken at various time points following vaccination and challenge 8 weeks later. The positive detection criterion is Ct ≤ 45. Vaccine-associated viremia was confirmed to be at undetectable levels in all animals starting 3–4 weeks prior to euthanasia.

T-2 Group (V5–V8): At 59 dpv (first challenge time point), the mean Ct value was 30.19 ± 1.84, while the Ct value at euthanasia (46 dpc) was 39.02 ± 0.45 (Figure 6a and Table S5). In contrast, serum Ct values at the first challenge time point averaged 43.72 ± 2.21, with undetectable ASF target gene levels (45.00 ± 0.00) at euthanasia (Figure 6b and Table S6). Oral swab Ct values at the first challenge time point averaged 45.00 ± 0.00, with very low ASF target gene levels (45.00 ± 0.00) confirmed at euthanasia (46 dpc) (Figure S3 and Table S7). Rectal swabs similarly showed extremely low values (45.00 ± 0.00) at euthanasia (Figure S4 and Table S8).

**Figure 7:**
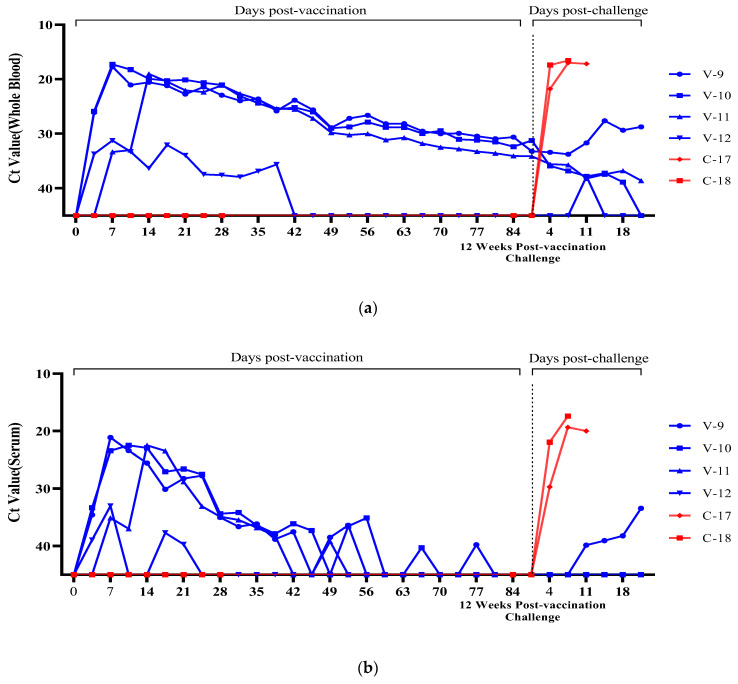
(**a**) This graph shows vaccine-associated viremia in whole blood following challenge at 12 weeks post-vaccination. The positivity threshold is Ct ≤ 45. Over time, vaccine-associated viremia shows a sharp decline, with two out of the four animals testing negative. (**b**) This graph shows vaccine-associated viremia observed in serum samples at different time points following vaccination and challenge 12 weeks later. The positive detection criterion is Ct ≤ 45. Starting 3 weeks prior to euthanasia, vaccine-associated viremia was confirmed to be at undetectable levels in all animals except one.

T-3 Group (V9–V12): At 87 dpv (first challenge time point), the mean Ct value was 35.89 ± 5.36, while the Ct value at euthanasia (21 dpc) was 39.33 ± 6.65 (Figure 7a and Table S9). As expected, serum Ct values at the first challenge time point averaged 45.00 ± 0.00, with very low ASF target gene levels (42.108 ± 5.01) confirmed at euthanasia (Figure 7b and Table S10). Oral swab Ct values at the first challenge time point averaged 45.00 ± 0.00, with very low ASF target gene levels (41.77 ± 3.23) confirmed at euthanasia (21 dpc) (Figure S5 and Table S11). Rectal swabs similarly showed low values (43.85 ± 1.98) at euthanasia (Figure S6 and Table S12).

Individual Animal Observations: Regarding vaccine-associated viremia results, individual-specific findings included notable observations for animal V-12, which showed ASF target gene detection in whole blood only until 38 dpv post-vaccination. No ASF target gene detection occurred post-challenge or even at euthanasia approximately 3 months post-vaccination. Remarkably, both oral and rectal samples from V-12 showed no viremia throughout the study period, including post-vaccination and post-challenge phases, distinguishing this animal from others in the study.

### 3.4. Evaluation of Serum Cytokines Against Multiple and Delayed Challenges Following ASFV-G-ΔI177L/ΔLVR Vaccination

Serum samples from pigs vaccinated against ASF and those receiving only the ASFV challenge were compared for cellular immune markers (IFN-γ and IL-12/IL-23 p40) and the anti-inflammatory cytokine IL-1ra, providing a broader perspective than pro-inflammatory cytokines alone. For reference, the cytokine analysis results in this study were limited by a small sample size, which constrained the reliability of *p*-value-based interpretation. The data are presented as mean ± standard deviation to illustrate the overall trends and variability rather than to imply statistical significance.

#### 3.4.1. Post-Vaccination Serum Cytokine Responses

**Figure 8:**
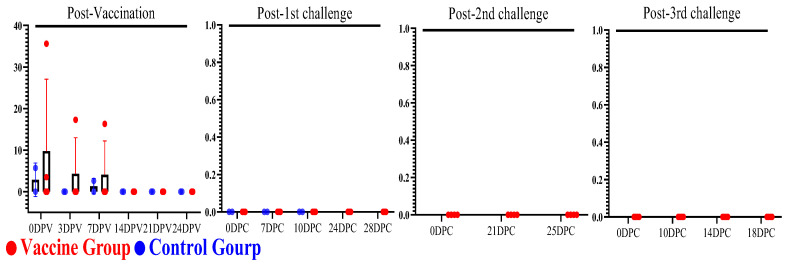
Interferon(IFN)-γ ELISA. A temporary increase was observed in some individuals during the initial phase of vaccination, but no consistent overall upward trend was evident. Following the challenge, a similar pattern persisted without any significant additional increase. DPV, days post-vaccination; DPC, days post-challenge.

For IFN-γ, transient increases were observed in some animals during the early vaccination period; however, there was substantial inter-individual variation overall, and no distinct consistent elevation pattern was apparent. Additionally, no significant differences between groups were observed throughout the post-vaccination period, and similar patterns were maintained without notable additional elevation following challenge inoculation (Figure 8).

**Figure 9:**
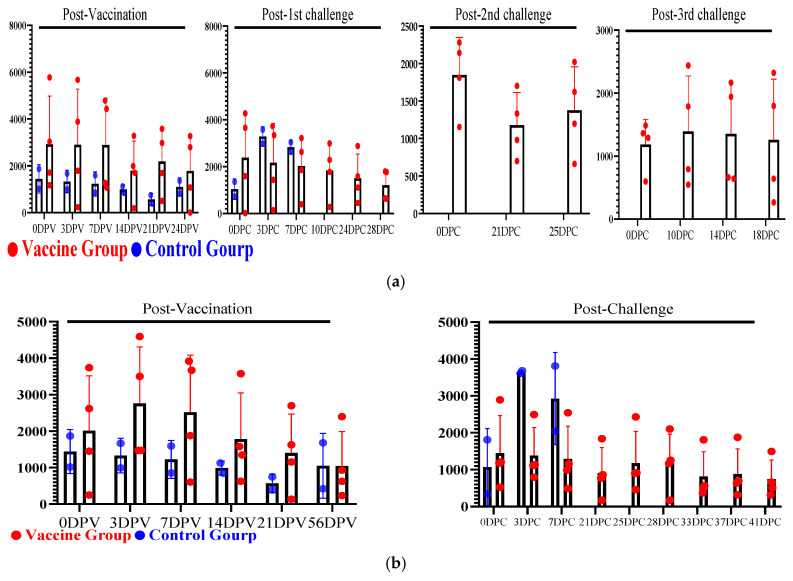

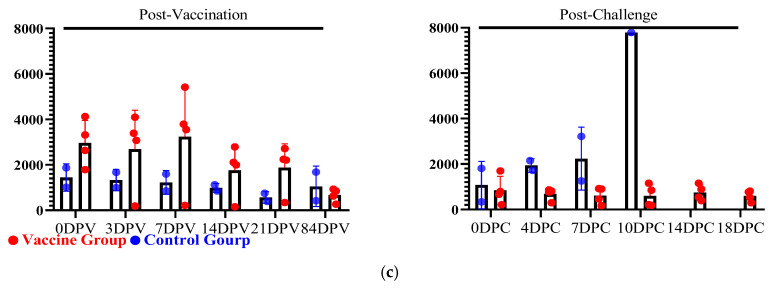
(**a**) IL-12/IL-23p40 ELISA. T-1 Group: The IL-12/IL-23p40 response showed an initial increase 7–14 days after vaccination, followed by a gradual stabilization trend. Even after repeated challenge vaccinations, the overall IL-12/IL-23p40 response pattern remained largely unchanged and maintained stabilization. C-1 Group: A sharp increase was observed 7 days after challenge vaccination. Subsequently, all control animals died. (**b**) IL-12/IL-23p40 ELISA. T-2 Group: In the group challenged 8 weeks after vaccination, a gradual stabilization trend was observed following an initial rise between days 7 and 14 immediately after vaccination. A similar trend was confirmed post-challenge without significant changes. C-2 Group: A sharp rise was observed on day 7 after challenge vaccination. Subsequently, all control animals died. (**c**) IL-12/IL-23p40 ELISA. T-3 Group: The group challenged at 12 weeks post-vaccination showed a trend of initial increase from 7 to 14 days after vaccination, followed by gradual stabilization. A similar trend was observed post-challenge without significant changes. C-3 Group: A sharp increase was observed on day 7 after challenge vaccination. Subsequently, all control animals died.

For IL-12/IL-23 p40, the T-1 group showed initial elevation followed by gradual stabilization, with levels of 2921.7 ± 2057.8 pg/mL immediately post-vaccination, 2892.8 ± 1989.8 pg/mL at day 7, and 1790.9 ± 1269.7 pg/mL at day 14 (Figure 9a). The T-2 group exhibited similar patterns, with levels of 2015.2 ± 1502.3 pg/mL immediately post-vaccination, 2517.2 ± 1566.2 pg/mL at day 7, and 1782.6 ± 1263.3 pg/mL at day 14 (Figure 9b). The T-3 group also demonstrated initial elevation followed by stabilization, showing 2965.0 ± 992.0 pg/mL immediately post-vaccination, 3243.1 ± 2181.9 pg/mL at day 7, and 1764.0 ± 1127.6 pg/mL at day 14 (Figure 9c).

**Figure 10:**
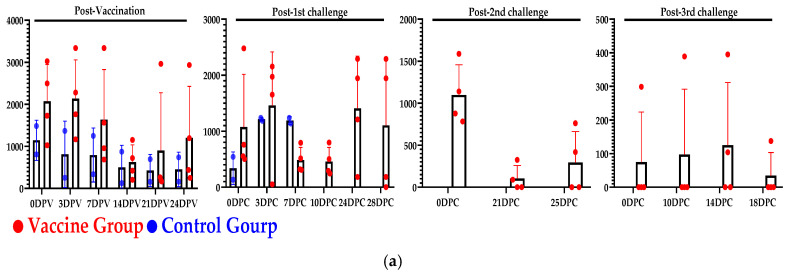

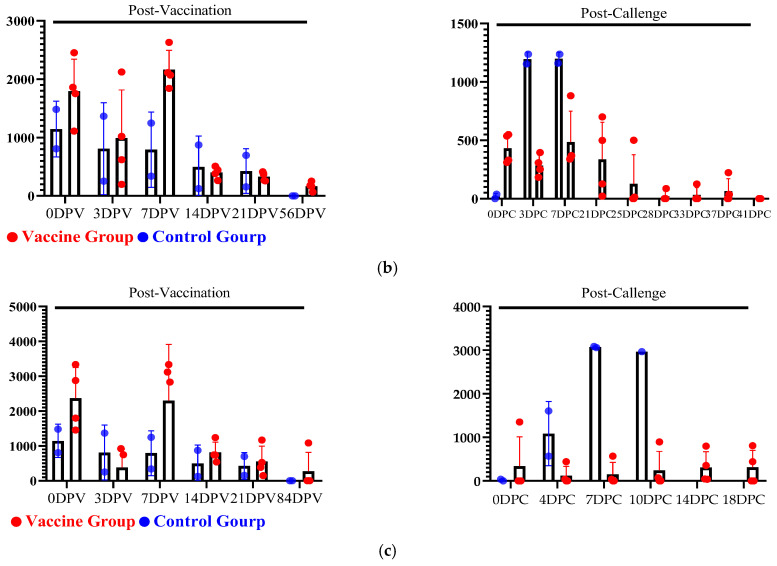
(**a**) Interleukin-1 receptor antagonist (IL-1ra) ELISA. T-1 Group: The IL-1ra response showed an initial increase 7–14 days after vaccination, followed by a gradual stabilization trend. Even after repeated challenge vaccinations, the overall IL-1ra response pattern remained largely unchanged and maintained stabilization. C-1 Group: A sharp increase was observed 7 days after challenge vaccination. Subsequently, all control animals died. (**b**) IL-1ra ELISA. T-2 Group: The group challenged at 8 weeks post-vaccination showed an initial rise immediately after vaccination, followed by a gradual stabilization trend. A similar trend was observed without significant change even after the challenge. C-2 Group: A sharp increase was observed on day 7 post-challenge vaccination. Subsequently, all control animals died. (**c**) IL-1ra ELISA. T-3 Group: The group challenged at 12 weeks post-vaccination showed a trend of initial increase from 7 to 14 days immediately after vaccination, followed by gradual stabilization. Post-challenge, a similar stabilization trend was observed without significant change. C-3 Group: A sharp increase was observed at 7 days post-challenge vaccination. Subsequently, all control animals died.

IL-1ra exhibited similar patterns. The T-1 group showed 2069.2 ± 875.4 pg/mL immediately post-vaccination, 1635.4 ± 1191.9 pg/mL at day 7, and 625.0 ± 411.3 pg/mL at day 14 (Figure 10a). The T-2 group demonstrated 1794.4 ± 549.2 pg/mL, 2165.0 ± 332.5 pg/mL, and 400.3 ± 104.5 pg/mL, respectively (Figure 10b). The T-3 group also displayed initial elevation followed by stabilization with levels of 2367.3 ± 885.6 pg/mL immediately post-vaccination, 2297.3 ± 1612.1 pg/mL at day 7, and 814.8 ± 297.2 pg/mL at day 14 (Figure 10c).

#### 3.4.2. Post-Challenge Serum Cytokine Responses

In the vaccinated groups (T-1, T-2 and T-3), no significant additional elevation of IL-12/IL-23 p40 or IL-1ra was observed following challenge inoculation, and levels remained similar to pre-challenge patterns. Notably, even after repeated challenges (1st/2nd/3rd) in the T-1 group, the overall cytokine response patterns did not substantially change and showed similar trends to the delayed challenge groups T-2 and T-3. Specifically, mean IL-12/IL-23 p40 concentrations in the T-1 group following the 1st, 2nd, and 3rd challenges were 2024.8 ± 1223.0 pg/mL, 1376.0 ± 582.3 pg/mL, and 1392.3 ± 880.9 pg/mL, respectively (Figure 9a). Similar trends were confirmed in the T-2 and T-3 groups, with post-challenge levels of 1295.2 ± 880.3 pg/mL and 612.3 ± 361.7 pg/mL, respectively, showing minimal variation (Figure 9b,c).

IL-1ra also demonstrated relatively stable patterns in vaccinated groups without significant additional elevation following challenge. In the T-1 group, levels remained unchanged even after repeated challenges at 484.8 ± 226.5 pg/mL, 103.7 ± 153.5 pg/mL, and 97.2 ± 194.5 pg/mL, respectively (Figure 10a). The T-2 and T-3 groups also maintained similar levels to pre-challenge conditions with post-challenge values of 486.2 ± 264.0 pg/mL and 151.1 ± 278.1 pg/mL, respectively (Figure 10b and Figure 10c).

In contrast, the control groups (C-1, C-2, C-3) commonly exhibited rapid elevation of both IL-12/IL-23 p40 and IL-1ra following challenge inoculation. Representatively, IL-12/IL-23 p40 in control groups increased from 1038.4 ± 439.2 pg/mL immediately pre-challenge to 2836.0 ± 291.4 pg/mL at day 7 post-challenge (Figure 9a–c), while IL-1ra similarly increased from 338.7 ± 290.3 pg/mL to 1188.4 ± 69.9 pg/mL at the same time point (Figure 10a–c). Subsequently, control animals showed mortality trends, and these responses were consistently confirmed in control groups at each challenge time point.

In vaccinated groups, limited and controlled elevation responses of IL-12/IL-23 p40 and IL-1ra occurred during early vaccination followed by stabilization, with no excessive cytokine elevation induced following challenge inoculation. Conversely, control groups showed rapid elevation of both markers following challenge, which was interpreted as a pattern associated with fatal clinical progression.

## 4. Discussion

Despite over a century of research since its first identification in 1921, ASFV continues to present formidable challenges that evade conventional eradication strategies. While achieving complete sterile immunity through vaccination has proven elusive, the ongoing devastating socioeconomic impact on the global swine industry necessitates a pragmatic shift in our approach. Given the urgent need to address food security concerns, the strategic deployment of safety-validated LAVs should be prioritized based on regional endemicity patterns and national policy frameworks. This approach represents a more realistic alternative to delaying critical interventions while pursuing an idealized, yet currently unattainable, universal vaccine solution.

Therefore, the development of safe and effective vaccines against ASF represents the most critical challenge for the global swine industry, particularly in endemic regions of Asia where smallholder livestock farming coexists with high viral pressure [11,21,28,29]. This study evaluated the efficacy and immunogenicity of multiple-gene-deletion LAV candidates under experimental conditions mimicking these high-risk environments, with particular focus on repeated and delayed exposure to homologous wild-type ASFV.

One of the most notable findings of this study was the observation of complete protection, evidenced by a 100% survival rate and the absence of clinical symptoms across all vaccinated groups, despite rigorous challenge protocols. While previous studies have demonstrated the initial protective efficacy of LAVs [14,18,26], our results extend these findings by confirming that immune responses remain robust even after three consecutive challenges. This “cumulative viral pressure” model is highly relevant to endemic regions where pigs are continuously exposed to the virus due to environmental contamination, mechanical transmission via vehicles and personnel, and proximity to infected backyard farms. Furthermore, delayed challenges conducted at 8 and 12 weeks post-vaccination demonstrated that vaccine-induced immunity does not decline rapidly, suggesting that a single vaccination in growing pigs could potentially provide protection against ASF until market age. This confirms the provision of sustained protection essential for long-term field application.

Although this was not a field trial in a real small-scale farm environment with typical pathogen loads, all experiments were conducted under conditions designed to validate vaccine efficacy with minimal potential for immunological interference.

A primary concern following LAV is vaccine virus shedding that could affect cohabiting animals and the surrounding environment. This shedding may involve vaccine genes or viruses remaining detectable in blood, oral secretions, and fecal samples. Importantly, in this study, progressive evidence of partial reduced viral replication was observed following vaccination and challenge. Despite three consecutive challenges at 28-day intervals, minimal levels of vaccine genome were detected only in whole blood samples from one out of four animals, while no detection was observed in blood, oral, and fecal samples from all other animals. This suggests that the vaccine-induced immune system effectively prevents pathogen survival and replication within the host, a characteristic of controlled viral residue that provides additional safety assurance for field applications.

The near-absence of viral shedding under repeated high-dose challenges indicates that vaccinated animals not only resist clinical disease but also limit secondary transmission potential. This dual protection mechanism represents critical advancement toward population-level disease control, particularly significant in endemic regions where continuous viral circulation threatens both vaccinated and unvaccinated populations.

Although partial, reduced viral replication demonstrates that LAV-established immune memory is sufficiently robust for rapid neutralization upon subsequent viral exposure, supporting the potential for broader epidemiological control beyond individual animal protection.

A key observation from the clinical trial was the differential pattern between vaccine genome detection and antibody maintenance. While the majority of vaccinated pigs showed persistent detection of the vaccine genome in blood alongside high antibody titers, some individuals maintained high antibody levels for up to 4 months even after the vaccine genome was completely cleared or significantly reduced. This suggests that initial replication of the LAV strain alone is sufficient to induce long-term memory T-cell and B-cell responses. From a safety perspective, the host’s ability to ultimately clear the vaccine virus while maintaining protective immunity is a highly desirable characteristic, reducing the risk of chronic persistence or potential virulence reversion in the field.

To understand the cellular mechanisms underlying this robust protection, we analyzed the profiles of key pro-inflammatory and anti-inflammatory cytokines. According to previous reports, sustained levels of IFN-γ and IL-12/23 p40 subunit in protected pigs indicate a strong Th1-biased cellular immune response. IL-12 and IL-23 play pivotal roles in linking innate and adaptive immunity, promoting the differentiation of IFN-γ-producing T cells and natural killer cells that are crucial for suppressing ASFV replication [22]. Although the high variability of the cytokine response limits the ability to draw definitive conclusions regarding the immune mechanism, we interpreted the overall trends as follows.

The cytokine analysis provides important insights into the immunological safety profile of the ASFV-G-ΔI177L/ΔLVR vaccine candidate. The controlled cytokine response pattern observed in this study clearly distinguishes the vaccine from wild-type ASFV infection, which is characterized by uncontrolled cytokine storms and hemorrhagic fever.

The transient IFN-γ elevation without sustained inflammatory responses demonstrates appropriate immune activation followed by effective regulation. Similarly, the IL-12/IL-23 p40 pattern showing maximum elevation immediately post-vaccination followed by stabilization by day 14 indicates controlled macrophage activation essential for cellular immunity against ASFV without prolonged inflammation.

Importantly, the balanced IL-1ra expression represents a critical safety mechanism of this vaccine candidate. IL-1ra effectively counteracts pro-inflammatory IL-1β, preventing the excessive systemic inflammation typically associated with live attenuated vaccines. This regulatory balance explains the absence of clinical side effects and creates a controlled immune environment that neutralizes the virus without triggering the fatal cytokine storm characteristic of virulent ASFV. These findings demonstrate that the ASFV-G-ΔI177L/ΔLVR vaccine achieves optimal immune activation while maintaining safety through effective cytokine regulation, supporting its potential as a viable ASF vaccine candidate. A reversion-to-virulence study is currently underway to further evaluate the safety of this vaccine candidate. The results will provide critical insights into its genetic stability, the demonstration of which will ultimately validate the vaccine’s overall safety profile.

Furthermore, from a vaccine virus shedding perspective, the controlled excretion of the vaccine virus through oral and fecal routes, as observed in swab examinations, further supports the field applicability of this vaccine candidate. In many Asian countries where backyard farms are scattered among smallholder operations with suboptimal biosecurity, a vaccine that provides broad protection while limiting shedding is an essential tool. Our “repeated and delayed re-infection” model serves as a strategic evaluation framework for assessing vaccine durability in this specific geographical context.

## 5. Conclusions

The multiple-gene-deletion LAV candidate demonstrated exceptional durability and safety in high-intensity challenge models. The LAV used in this study was administered to pathogen-free animals selected under controlled conditions to establish a baseline for vaccine efficacy without immunological interference; however, these standardized experimental conditions were essential for isolating vaccine-specific immune responses and establishing basic safety and efficacy indicators that would serve as the foundation for future field trials conducted in more complex environments involving natural pathogen exposure. Importantly, the present study was designed exclusively as a homologous challenge trial, demonstrating that the ASFV-G-ΔI177L/ΔLVR vaccine candidate provides robust protection against homologous ASFV strains; heterologous challenge experiments were not conducted or evaluated in this investigation. This vaccine candidate achieved safety by inducing limited and controlled immune responses during the early vaccination phase while effectively suppressing the fatal cytokine surge observed in control groups upon actual viral exposure, thereby enhancing survival rates. This strongly supports that vaccine-induced immune memory suppresses wild-type virus replication and controls the host immune system to prevent runaway responses. Therefore, the results from the repeated and delayed homologous challenge experiments conducted in this study provide essential foundational data supporting continued development of this vaccine candidate. Despite these promising findings, we acknowledge several limitations in the present study: the small experimental group size, the exclusive use of a homologous virus, the low number of passages used for genome stability analysis, and the fact that whole-genome sequencing was not performed after co-infection. Future research must address all these limitations; additional studies, including larger experimental cohorts, heterologous strain evaluations, extended serial passage trials accompanied by whole-genome sequencing, and field trials, will be necessary before regulatory consideration in accordance with international guidelines.

## Figures and Tables

**Table 1:** Summary of the ASFV vaccine clinical trial design.

Trial Group	Animal No. Identification	Challenge Timing		Vaccine/Dose	Challenge/Dose	Route
T-1	4 (* V1–V4)	1st Challenge	4 WPV	ASFV-G-ΔI177L/ΔLVR/10^3^TCID_50_	ASFV-Hwacheon/2020 (G-II)/10^2^HAD_50_	IM
		2nd Challenge	8 WPV			
		3rd Challenge	12 WPV			
T-2	4 (V5–V8)	Challenge	8 WPV			
T-3	4 (V9–V12)	Challenge	12 WPV			
C-1	2 (^§^ C13–C14)	T-1 Challenge control		Not applicable		
C-2	2 (C15–C16)	T-2 Challenge control				
C-3	2 (C17–C18)	T-3 Challenge control				

*: Vaccinated, ^§^: Control, WPV: week post-vaccination, IM: Intramuscular.

**Table 2:** ASF vaccine antibody in blood samples of the T-1 and C-1 groups.

Group		S/N % (cELISA)																													
		DPV (Days Post-Vaccination)								1st DPC (1st Days Post-Challenge)									2nd DPC (2nd Days Post-Challenge)								3rd DPC (3rd Days Post-Challenge)				
		0	3	7	10	14	21	24	28	3	7	10	14	17	21	24	28	31	4	7	11	14	18	21	25	28	4	7	11	14	18
**T1**	**V-1**	75.6	73.5	35.9	25.2	23.7	24.1	25.9	22.5	20.3	15.0	10.6	8.4	6.8	4.6	3.0	1.0	0.1	0.6	0.6	0.6	1.0	0.6	1.4	1.2	1.6	1.7	0.6	0.0	0.3	0.9
	**V-2**	82.0	76.9	40.2	10.3	14.8	20.6	22.5	20.3	21.0	16.4	12.4	10.1	9.6	7.0	5.1	4.1	3.1	2.2	0.7	0.7	0.8	−0.5	0.0	−0.5	−0.6	−1.0	−1.8	−1.7	−1.6	−0.9
	**V-3**	85.1	73.6	51.3	28.0	26.5	24.9	27.5	18.6	13.7	8.5	4.3	3.1	2.6	1.5	1.7	0.6	1.4	1.0	0.9	0.9	0.9	0.7	1.2	0.5	1.4	0.6	0.2	0.2	−0.2	1.1
	**V-4**	76.9	63.3	47.0	22.4	20.4	19.6	22.5	18.1	11.4	11.6	11.0	8.7	7.9	5.9	.8	4.1	4.0	2.5	2.2	2.1	1.5	1.4	1.9	1.9	2.3	1.9	1.9	1.2	1.4	3.0
**C-1**	**C-13**	79.8	75.1	76.5	73.2	85.9	79.2	88.0	94.6	85.8	41.0	16.6	D	-	-	-	-	-	-	-	-	-	-	-	-	-	-	-	-	-	-
	**C-14**	76.9	81.2	77.5	73.3	83.7	70.8	92.1	87.5	73.2	29.6	20.7	D	-	-	-	-	-	-	-	-	-	-	-	-	-	-	-	-	-	-

**Table 3:** ASF vaccine antibody in blood samples of the T-2 and C-2 groups.

Group		S/N % (cELISA)																														
		DPV (Days Post-Vaccination)																		DPC (Days Post-Challenge)												
		0	3	7	10	14	17	21	24	28	31	35	38	42	45	49	52	56	59	4	7	11	14	18	21	25	28	32	35	39	42	46
**T2**	**V-5**	83.9	80.7	55.5	22.0	24.2	28.6	24.3	31.5	27.2	17.1	14.3	11.0	9.3	7.9	5.6	4.3	2.6	3.3	1.1	1.3	1.1	0.0	−0.6	0.0	−0.5	−0.7	−0.2	−0.7	−1.6	−1.8	1.4
	**V-6**	80.9	83.0	41.3	19.5	18.6	14.9	13.3	18.1	18.6	12.6	13.0	12.2	10.9	12.1	10.3	9.9	8.2	7.7	6.2	4.4	0.6	−0.3	−0.5	−0.7	−1.7	−1.1	0.2	−1.0	−1.4	−1.5	−1.2
	**V-7**	79.0	76.2	36.4	24.4	32.8	29.9	29.3	27.2	18.7	15.6	15.5	12.4	11.7	12.5	10.8	11.2	10.9	12.9	9.3	13.1	7.3	5.5	3.9	2.5	−0.4	0.3	0.5	−0.6	−0.1	−1.2	0.0
	**V-8**	82.9	75.6	48.3	24.0	20.3	18.4	19.2	19.9	14.7	10.9	10.7	7.7	6.9	6.8	4.6	3.8	3.3	4.0	2.2	1.8	0.1	−0.5	−0.9	−0.8	−1.7	−1.7	−1.9	−2.5	−2.0	−2.8	−2.2
**C-2**	**C-15**	82.2	88.0	85.3	78.4	78.8	82.3	83.8	85.0	89.9	No Data						83.9	89.7	82.8	89.7	48.6	D	-	-	-	-	-	-	-	-	-	-
	**C-16**	77.6	78.3	79.9	85.3	75.4	80.2	79.5	85.5	95.5							92.5	91.3	85.6	81.3	47.8	D	-	-	-	-	-	-	-	-	-	-

**Table 4:** ASF vaccine antibody in blood samples of the T-3 and C-3 groups.

Group		S/N % (cELISA)																															
		DPV (Days Post-Vaccination)																										DPC (Days Post-Challenge)					
		0	3	7	10	14	17	21	24	28	31	35	38	42	45	49	52	56	59	63	66	70	73	77	80	84	87	4	7	11	14	18	21
**T3**	**V-9**	83.0	74.8	37.9	17.2	16.4	15.5	13.9	12.8	9.3	6.4	6.9	4.3	3.6	3.9	3.0	1.9	1.3	1.9	1.2	1.4	1.1	0.8	0.4	0.5	0.5	0.6	0.5	−0.2	−0.6	−1.6	−2.7	−2.3
	**V-10**	87.6	75.2	52.9	27.9	30.1	29.4	32.9	32.7	20.5	11.9	10.7	7.3	6.3	5.9	5.8	4.0	4.2	4.4	3.6	3.5	2.6	2.6	2.1	2.1	2.7	2.8	3.4	1.5	1.3	1.5	2.0	2.0
	**V-11**	81.1	72.8	74.3	56.2	41.9	25.9	23.3	24.6	22.5	15.7	11.7	9.3	7.6	6.3	5.2	3.5	2.4	2.5	1.2	3.3	0.0	−0.2	−0.4	−0.5	−0.6	−0.5	−0.2	−1.5	−1.7	−1.7	−1.3	−1.7
	**V-12**	77.9	76.0	58.1	41.9	44.1	44.1	35.0	33.4	32.6	23.5	20.6	17.9	16.0	15.8	13.9	11.2	9.1	9.5	7.5	7.3	6.0	5.3	6.5	5.3	5.1	5.5	6.3	4.7	3.8	3.8	2.7	1.7
**C-3**	**C-17**	85.4	81.3	85.5	86.4	75.8	82.0	79.2	82.8	89.3	No Data														56.9	63.6	61.2	60.6	22.3	D	-	-	-
	**C-18**	79.3	84.1	85.5	79.6	82.7	90.9	90.4	89.3	102.3															81.0	56.5	83.6	60.9	D	-	-	-	-

Abbreviations: ASF, African swine fever; S/N %, sample-to-negative control percentage; DPV, days post-vaccination; DPC, days post-challenge. Values are presented as individual competitive enzyme-linked immunosorbent assay (cELISA) S/N% results. According to the assay criteria, S/N% values of ≤40% were considered positive, 41–49% doubtful, and ≥50% negative. “D” indicates that the animal died. “No Data” indicates that the sample was not collected or that the result was unavailable. “-“ indicates no available data.

## Data Availability

Data are contained within the article and Supplementary Materials.

## Supplementary Materials

The following supporting information can be downloaded at: https://www.mdpi.com/article/10.3390/vaccines14070561/s1, Tables S1–S4: Summarize qPCR Ct values for samples (whole blood, serum, oral, and rectal) from experimental groups T-1 and C-1 following vaccination and challenge infection. Tables S5–S8: Summarize qPCR Ct values for samples (whole blood, serum, oral, and rectal) from experimental groups T-2 and C-2 following vaccination and challenge infection. Tables S9–S12: Summarize qPCR Ct values for samples (whole blood, serum, oral, and rectal) from experimental groups T-3 and C-3 following vaccination and challenge infection. Figures S1 and S2: qPCR Ct values for oral and rectal samples from experimental groups T-1 and C-1 following vaccination and challenge infection. Figures S3 and S4: qPCR Ct values for oral and rectal samples from experimental groups T-2 and C-2 following vaccination and challenge infection. Figures S5 and S6: qPCR Ct values for oral and rectal samples from experimental groups T-3 and C-3 following vaccination and challenge infection.

## Abbreviations

The following abbreviations are used in this manuscript:

ASFV	African swine fever virus
LAVs	Live attenuated vaccines
WOAH	World Organization for Animal Health
PIPEC	Plum Island porcine epithelial cells
dpv	Days post-vaccination
dpc	Days post-challenge
WPV	Week post-vaccination
IM	Intramuscular
qPCR	Quantitative real-time PCR
HAD	Hemadsorption dose
TCID	Tissue culture infectious dose
ELISA	Enzyme-linked immunosorbent assay
Ct	Cycle threshold
