# Supplementary material for: A Repeated and Delayed Homologous Challenge Study Evaluating the Durability of Protection Induced by the Live Attenuated ASF Vaccine Candidate ASFV-G-ΔI177L/ΔLVR
Source: Vaccines (Basel). 2026 Jun 25;14(7):561. doi: 10.3390/vaccines14070561 (PMC13419286; doi:10.3390/vaccines14070561)
Supplement: Supplementary file 1 [file vaccines-14-00561-s001.zip › vaccines-4343048-supplementary.pdf]

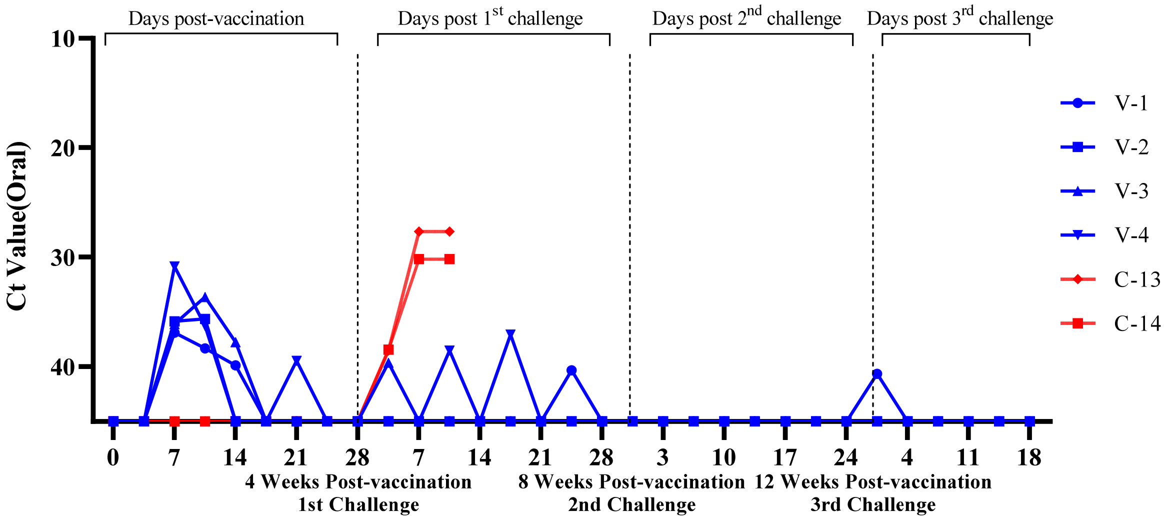

Figure S1. This graph shows vaccine-associated viremia observed in the oral swab after three repeated challenge experiments conducted 4 weeks post-vaccination. The positive detection criterion was cycle threshold (Ct) ≤45. From the third challenge until euthanasia, vaccine-associated viremia was confirmed negative in all animals.

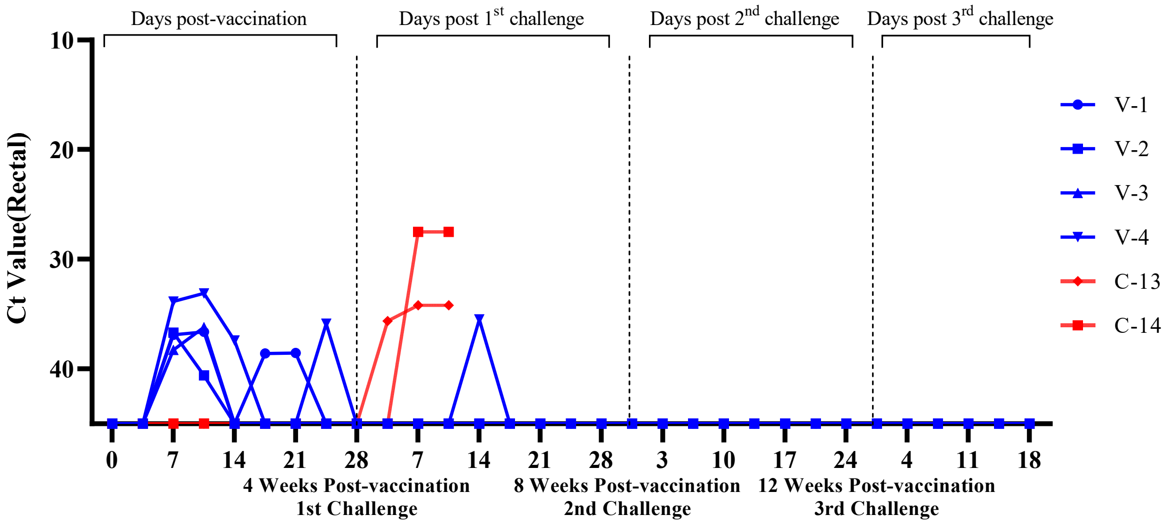

Figure S2. This graph shows vaccine-associated viremia observed in rectal swabs following three repeated challenge experiments conducted 4 weeks after vaccination. The positive detection criterion is C t≤45. No vaccine-associated viremia was observed after the second challenge.

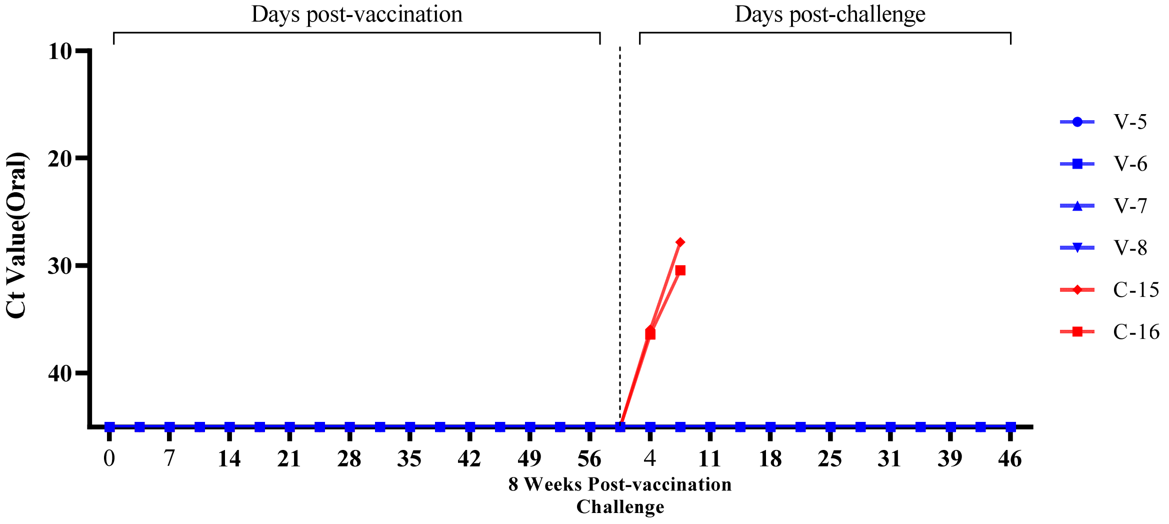

Figure S3. This graph shows the challenge performed at 8 weeks post-vaccination. It depicts the detection of vaccine-associated viremia in oral swabs thereafter. The positive detection criterion is Ct ≤45. All animals tested negative for vaccine-associated viremia until euthanasia following the challenge.

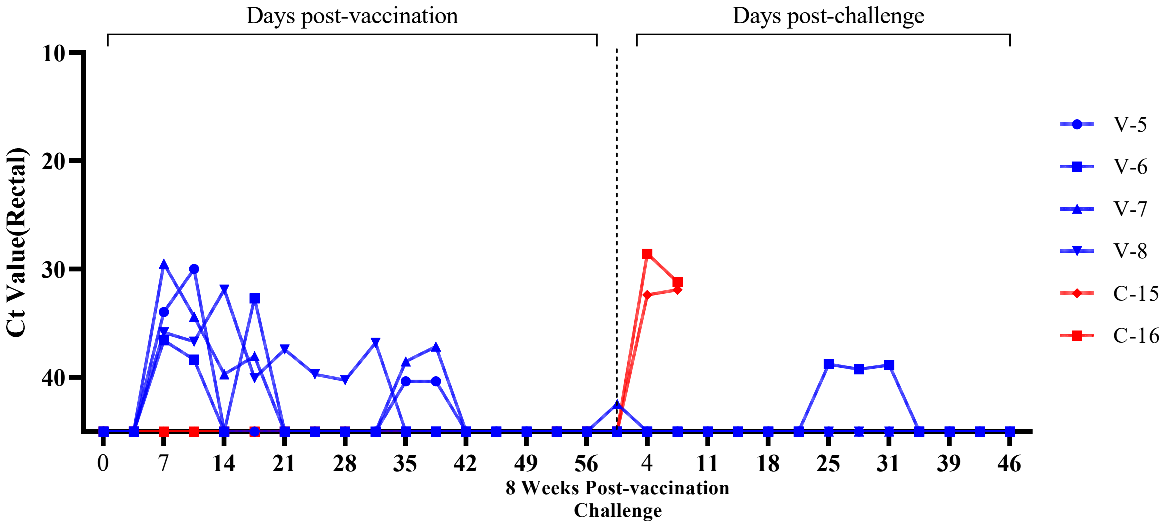

Figure S4. This graph shows the results of challenge experiments performed 8 weeks after vaccination. It indicates the detection of vaccine-related viremia in rectal swab specimens. The positive threshold is Ct ≤45. All animals remained negative for vaccine-related viremia from the time of infection until euthanasia.

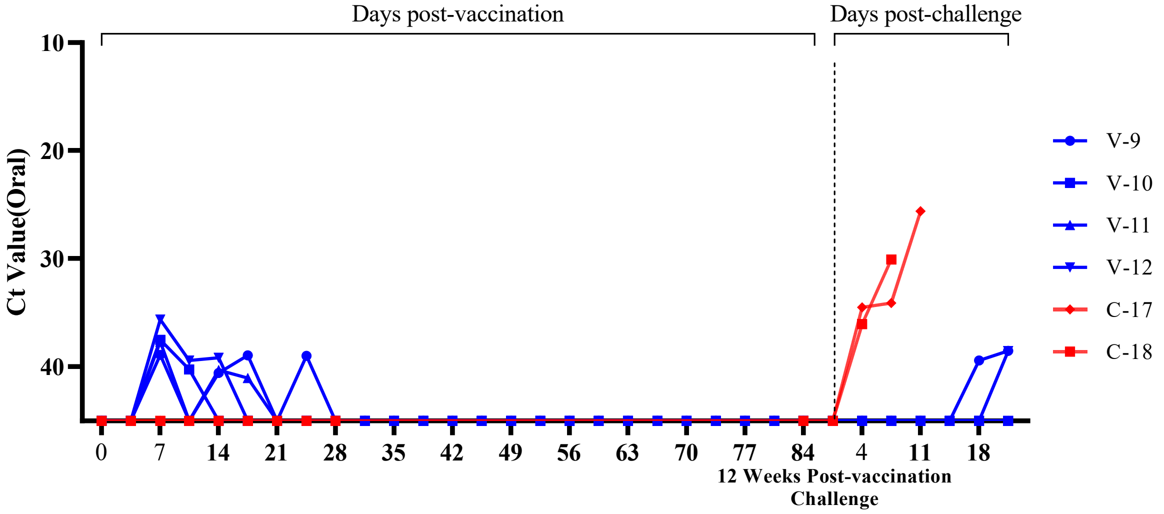

Figure S5. This graph shows the challenge performed at 12 weeks post-vaccination. It depicts the detection of vaccine-associated viremia in oral swabs thereafter. The positive detection criterion is Ct ≤45. Except for one animal, vaccine-associated viremia was confirmed negative in all animals until euthanasia following the challenge.

.

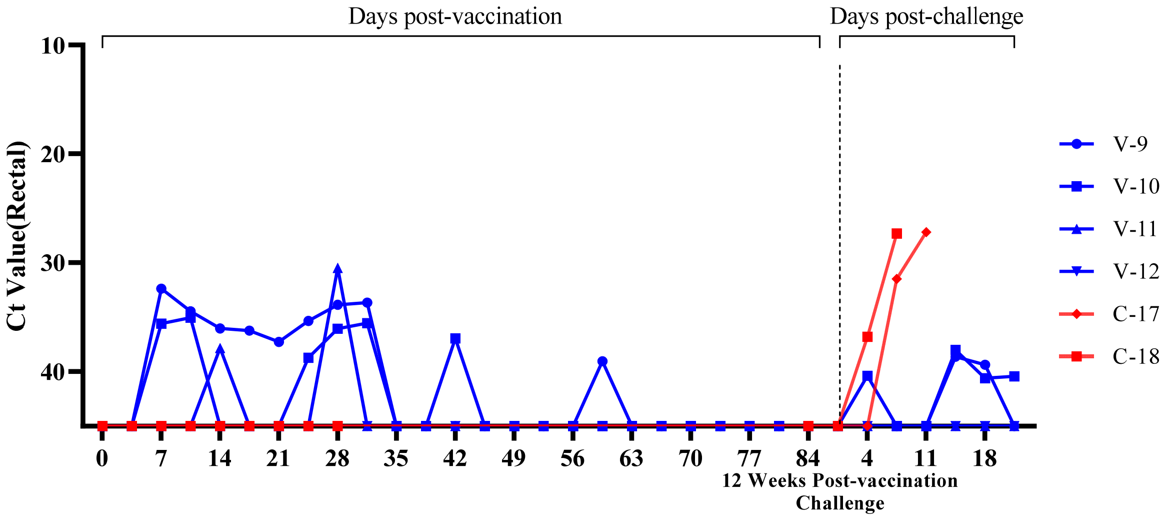

Figure S6. This graph shows the results of challenge experiments performed 12 weeks after vaccination. It indicates the detection of vaccine-related viremia in rectal swab specimens. The positive threshold is Ct ≤45. Most animals remained negative for vaccine-related viremia until euthanasia.

Supplementary Table S1. Individual qPCR Ct values in whole blood samples from the T-1 and C-1 groups.

Group		Ct (qPCR, Whole Blood)																														
		DPV (Days Post-Vaccination)									1st DPC (1st Days Post-Challenge)									2nd DPC (2nd Days Post-Challenge)								3rd DPC (3rd Days Post-Challenge)				
		**0**	**3**	**7**	**10**	**14**	**17**	**21**	**24**	**28**	**3**	**7**	**10**	**14**	**17**	**21**	**24**	**28**	**31**	**4**	**7**	**11**	**14**	**18**	**21**	**25**	**28**	**4**	**7**	**11**	**14**	**18**
T-1	V-1	45.00	21.37	18.44	18.64	20.36	19.30	20.95	22.28	22.24	22.45	22.83	23.91	23.64	25.63	26.10	26.95	25.50	26.98	28.25	27.09	29.47	30.28	29.12	30.84	31.96	32.55	35.14	36.97	36.63	37.70	38.93
	V-2	45.00	32.14	21.14	21.88	23.20	24.05	24.65	26.19	28.28	30.54	31.07	32.76	31.82	33.92	36.20	35.71	36.70	36.09	36.35	37.40	37.10	38.45	39.41	39.00	45.00	40.13	45.00	45.00	45.00	45.00	45.00
	V-3	45.00	29.15	21.58	21.89	22.89	25.46	25.89	27.63	28.82	31.67	30.92	31.95	31.33	33.00	35.26	34.84	35.78	26.87	35.49	36.44	37.67	37.81	37.76	39.51	38.49	39.90	45.00	45.00	45.00	45.00	45.00
	V-4	45.00	25.30	17.13	18.08	20.13	19.79	19.60	20.53	21.36	21.75	22.99	24.11	23.02	25.15	27.05	26.95	26.43	28.62	28.41	28.54	28.77	29.61	30.41	31.58	32.81	34.41	36.71	38.33	38.87	39.57	45.00
C-1	C-13	45.00	45.00	45.00	45.00	45.00	45.00	45.00	45.00	45.00	18.43	16.50	18.54	D	-	-	-	-	-	-	-	-	-	-	-	-	-	-	-	-	-	-
	C-14	45.00	45.00	45.00	45.00	45.00	45.00	45.00	45.00	45.00	24.33	16.89	17.19	D	-	-	-	-	-	-	-	-	-	-	-	-	-	-	-	-	-	-

Supplementary Table S2. Individual qPCR Ct values in serum samples from the T-1 and C-1 groups.

Group		Ct (qPCR, Serum)																														
		DPV (Days Post-Vaccination)									1st DPC (1st Days Post-Challenge)									2nd DPC (2nd Days Post-Challenge)								3rd DPC (3rd Days Post-Challenge)				
		**0**	**3**	**7**	**10**	**14**	**17**	**21**	**24**	**28**	**3**	**7**	**10**	**14**	**17**	**21**	**24**	**28**	**31**	**4**	**7**	**11**	**14**	**18**	**21**	**25**	**28**	**4**	**7**	**11**	**14**	**18**
T-1	V-1	45.00	32.39	26.57	26.78	28.49	30.51	29.16	33.45	36.05	33.40	36.76	37.27	34.91	37.87	37.83	36.33	39.31	38.87	37.58	45.00	45.00	45.00	38.97	45.00	39.27	45.00	45.00	45.00	45.00	45.00	45.00
	V-2	45.00	38.47	27.87	30.74	31.42	32.42	33.11	38.35	45.00	45.00	45.00	45.00	45.00	45.00	45.00	45.00	45.00	30.08	45.00	45.00	45.00	45.00	45.00	45.00	45.00	45.00	45.00	45.00	45.00	45.00	45.00
	V-3	45.00	39.23	28.54	30.21	30.20	33.64	36.37	45.00	45.00	40.82	36.73	45.00	45.00	45.00	45.00	45.00	45.00	45.00	45.00	45.00	45.00	45.00	45.00	45.00	45.00	45.00	45.00	45.00	45.00	45.00	45.00
	V-4	45.00	35.41	23.20	26.55	28.17	27.23	30.40	31.39	33.59	33.61	35.73	36.12	36.26	36.66	37.69	33.45	33.60	35.85	38.72	38.09	39.05	45.00	39.07	45.00	45.00	45.00	45.00	45.00	45.00	45.00	45.00
C-1	C-13	45.00	45.00	45.00	45.00	45.00	45.00	45.00	45.00	45.00	26.36	19.40	24.17	D	-	-	-	-	-	-	-	-	-	-	-	-	-	-	-	-	-	-
	C-14	45.00	45.00	45.00	45.00	45.00	45.00	45.00	45.00	45.00	33.54	21.44	22.88	D	-	-	-	-	-	-	-	-	-	-	-	-	-	-	-	-	-	-

Supplementary Table S3. Individual qPCR Ct values in oral swab samples from the T-1 and C-1 groups.

Group		Ct (qPCR, Oral)																														
		DPV (Days Post-Vaccination)									1st DPC (1st Days Post-Challenge)									2nd DPC (2nd Days Post-Challenge)								3rd DPC (3rd Days Post-Challenge)				
		0	3	7	10	14	17	21	24	28	3	7	10	14	17	21	24	28	31	4	7	11	14	18	21	25	28	4	7	11	14	18
T-1	V-1	45.00	45.00	36.89	38.32	39.87	45.00	45.00	45.00	45.00	45.00	45.00	45.00	45.00	45.00	45.00	40.32	45.00	45.00	45.00	45.00	45.00	45.00	45.00	45.00	45.00	40.63	45.00	45.00	45.00	45.00	45.00
	V-2	45.00	45.00	35.84	35.62	45.00	45.00	45.00	45.00	45.00	45.00	45.00	45.00	45.00	45.00	45.00	45.00	45.00	45.00	45.00	45.00	45.00	45.00	45.00	45.00	45.00	45.00	45.00	45.00	45.00	45.00	45.00
	V-3	45.00	45.00	36.10	33.62	37.74	45.00	45.00	45.00	45.00	39.63	45.00	45.00	45.00	45.00	45.00	45.00	45.00	45.00	45.00	45.00	45.00	45.00	45.00	45.00	45.00	45.00	45.00	45.00	45.00	45.00	45.00
	V-4	45.00	45.00	30.82	36.26	45.00	45.00	39.45	45.00	45.00	45.00	45.00	38.50	45.00	37.07	45.00	45.00	45.00	45.00	45.00	45.00	45.00	45.00	45.00	45.00	45.00	45.00	45.00	45.00	45.00	45.00	45.00
C-1	C-13	45.00	45.00	45.00	45.00	45.00	45.00	45.00	45.00	45.00	38.52	27.65	27.65	D	-	-	-	-	-	-	-	-	-	-	-	-	-	-	-	-	-	-
	C-14	45.00	45.00	45.00	45.00	45.00	45.00	45.00	45.00	45.00	38.44	30.15	30.15	D	-	-	-	-	-	-	-	-	-	-	-	-	-	-	-	-	-	-

Supplementary Table S4. Individual qPCR Ct values in rectal samples from the T-1 and C-1 groups.

Group		Ct (qPCR, Fecal)																														
		DPV (Days Post-Vaccination)									1st DPC (1st Days Post-Challenge)									2nd DPC (2nd Days Post-Challenge)								3rd DPC (3rd Days Post-Challenge)				
		0	3	7	10	14	17	21	24	28	3	7	10	14	17	21	24	28	31	4	7	11	14	18	21	25	28	4	7	11	14	18
T-1	V-1	45.00	45.00	36.88	36.60	45.00	38.58	38.54	45.00	45.00	45.00	45.00	45.00	45.00	45.00	45.00	45.00	45.00	45.00	45.00	45.00	45.00	45.00	45.00	45.00	45.00	45.00	45.00	45.00	45.00	45.00	45.00
	V-2	45.00	45.00	36.69	40.58	45.00	45.00	45.00	45.00	45.00	45.00	45.00	45.00	45.00	45.00	45.00	45.00	45.00	45.00	45.00	45.00	45.00	45.00	45.00	45.00	45.00	45.00	45.00	45.00	45.00	45.00	45.00
	V-3	45.00	45.00	38.26	36.20	45.00	45.00	45.00	45.00	45.00	45.00	45.00	45.00	45.00	45.00	45.00	45.00	45.00	45.00	45.00	45.00	45.00	45.00	45.00	45.00	45.00	45.00	45.00	45.00	45.00	45.00	45.00
	V-4	45.00	45.00	33.83	33.11	37.40	45.00	45.00	35.88	45.00	45.00	45.00	45.00	35.48	45.00	45.00	45.00	45.00	45.00	45.00	45.00	45.00	45.00	45.00	45.00	45.00	45.00	45.00	45.00	45.00	45.00	45.00
C-1	C-13	45.00	45.00	45.00	45.00	45.00	45.00	45.00	45.00	45.00	35.62	34.18	34.18	D	-	-	-	-	-	-	-	-	-	-	-	-	-	-	-	-	-	-
	C-14	45.00	45.00	45.00	45.00	45.00	45.00	45.00	45.000	45.00	45.00	27.49	27.49	D	-	-	-	-	-	-	-	-	-	-	-	-	-	-	-	-	-	-

Abbreviations: Ct, cycle threshold; DPV, days post-vaccination; DPC, days post-challenge. Values are presented as individual qPCR Ct values for the ASFV p72 gene. According to the assay criteria, samples with Ct values <45 were considered positive, whereas Ct values of 45.000 were considered negative or undetermined at the assay cutoff. “D” indicates that the animal died. “-” indicates no available data.

Supplementary Table S5. Individual qPCR Ct values in whole blood samples from the T-2 and C-2 groups.

Group		Ct (qPCR, Whole Blood)																														
		DPV (Days Post-Vaccination)																		DPC (Days Post-Challenge)												
		0	3	7	10	14	17	21	24	28	31	35	38	42	45	49	52	56	59	4	7	11	14	18	21	25	28	4	7	11	14	18
T-2	V-5	45.00	26.81	18.23	20.01	20.96	20.95	21.90	22.64	23.91	25.35	26.86	28.03	27.39	29.53	30.76	31.66	30.86	32.64	32.67	32.38	33.47	34.24	34.47	35.18	35.40	36.08	39.33	39.18	45.00	45.00	39.56
	V-6	45.00	30.02	18.59	22.26	20.28	22.50	21.48	22.80	24.22	24.77	26.39	27.65	27.66	28.57	30.52	30.61	30.53	31.28	31.39	28.25	31.03	31.88	33.34	32.81	33.18	34.41	37.38	35.34	36.19	36.49	39.18
	V-7	45.00	26.09	17.77	18.45	18.75	21.19	20.22	19.29	21.96	22.02	23.75	23.94	24.98	26.38	28.15	28.09	28.49	28.41	28.95	29.06	30.49	31.06	31.87	32.07	32.40	33.14	37.07	36.44	37.46	38.20	39.02
	V-8	45.00	26.12	17.17	19.24	19.65	19.93	20.49	21.17	22.52	22.48	25.49	24.15	23.57	26.02	29.17	28.29	27.98	28.41	28.95	29.77	30.20	30.48	31.13	31.83	32.85	32.86	35.91	36.69	37.39	39.62	38.32
C-2	C-15	45.00	45.00	45.00	45.00	45.00	45.00	45.00	45.00	45.00	No Data						45.00	45.00	45.00	16.03	15.49	D	-	-	-	-	-	-	-	-	-	-
	C-16	45.00	45.00	45.00	45.00	45.00	45.00	45.00	45.00	45.00							45.00	45.00	45.00	16.18	15.80	D	-	-	-	-	-	-	-	-	-	-

Supplementary Table S6. Individual qPCR Ct values in serum samples from the T-2 and C-2 groups.

Group		Ct (qPCR, Serum)																														
		DPV (Days Post-Vaccination)																		DPC (Days Post-Challenge)												
		0	3	7	10	14	17	21	24	28	31	35	38	42	45	49	52	56	59	4	7	11	14	18	21	25	28	4	7	11	14	18
T-2	V-5	45.00	35.11	20.08	26.99	28.13	28.75	29.97	33.26	34.12	37.93	39.11	37.74	45.00	45.00	38.75	45.00	45.00	45.00	45.00	45.00	40.57	45.00	45.00	45.00	45.00	45.00	45.00	45.00	45.00	45.00	45.00
	V-6	45.00	39.21	28.15	27.78	30.38	31.93	32.59	34.52	37.83	36.84	45.00	45.00	45.00	45.00	38.70	45.00	45.00	45.00	45.00	37.13	39.83	39.69	40.36	38.96	39.72	45.00	45.00	45.00	45.00	45.00	45.00
	V-7	45.00	34.61	24.48	28.30	24.58	25.20	28.37	31.13	34.19	33.96	34.74	38.01	37.36	45.00	39.76	38.61	39.18	39.88	39.30	36.73	45.00	37.89	38.29	45.00	45.00	45.00	45.00	45.00	45.00	45.00	45.00
	V-8	45.00	33.74	18.88	24.68	27.37	26.87	27.80	31.98	31.99	35.30	36.25	38.77	38.78	40.30	45.00	39.39	40.12	45.00	38.49	45.00	45.00	45.00	39.84	45.00	45.00	39.24	45.00	45.00	45.00	45.00	45.00
C-2	C-15	45.00	45.00	45.00	45.00	45.00	45.00	45.00	45.00	45.00	No Data						45.00	45.00	45.00	19.96	18.27	D	-	-	-	-	-	-	-	-	-	-
	C-16	45.00	45.00	45.00	45.00	45.00	45.00	45.00	45.00	45.00							45.00	45.00	45.00	21.89	18.70	D	-	-	-	-	-	-	-	-	-	-

Supplementary Table S7. Individual qPCR Ct values in oral swab samples from the T-2 and C-2 groups.

Group		Ct (qPCR, Oral)																													
		DPV (Days Post-Vaccination)																		DPC (Days Post-Challenge)												
		0	3	7	10	14	17	21	24	28	31	35	38	42	45	49	52	56	59	4	7	11	14	18	21	25	28	4	7	11	14	18
T-2	V-5	45.00	45.00	38.85	37.19	45.00	40.55	45.00	45.00	45.00	45.00	45.00	45.00	45.00	45.00	45.00	45.00	45.00	45.00	45.00	45.00	45.00	45.00	45.00	45.00	45.00	45.00	45.00	45.00	45.00	45.00	45.00
	V-6	45.00	45.00	40.22	37.62	45.00	40.62	45.00	35.31	45.00	45.00	45.00	45.00	45.00	45.00	45.00	45.00	45.00	45.00	45.00	45.00	45.00	45.00	45.00	45.00	45.00	45.00	45.00	45.00	45.00	45.00	45.00
	V-7	45.00	45.00	39.10	36.95	45.00	45.00	39.25	39.77	41.12	45.000	45.00	45.00	45.00	45.00	45.00	45.00	45.00	45.00	45.00	45.00	45.00	35.87	45.00	45.00	45.00	45.00	45.00	45.00	39.94	45.00	45.00
	V-8	45.00	45.00	32.49	38.15	38.00	45.00	45.00	39.25	40.33	45.00	45.00	40.24	45.00	45.00	45.00	45.00	45.00	45.00	45.00	45.00	45.00	45.00	45.00	45.00	45.00	45.00	45.00	45.00	45.00	45.00	45.00
C-2	C-15	45.00	45.00	45.00	45.00	45.00	45.00	45.00	45.00	45.00			No Data				45.00	45.00	45.00	35.91	27.78	D	-	-	-	-	-	-	-	-	-	-
	C-16	45.00	45.00	45.00	45.00	45.00	45.00	45.00	45.00	45.00							45.00	45.00	45.00	36.39	30.40	D	-	-	-	-	-	-	-	-	-	-

Supplementary Table S8. Individual qPCR Ct values in rectal samples from the T-2 and C-2 groups.

Group		Ct (qPCR, Fecal)																													
		DPV (Days Post-Vaccination)																		DPC (Days Post-Challenge)												
		0	3	7	10	14	17	21	24	28	31	35	38	42	45	49	52	56	59	4	7	11	14	18	21	25	28	4	7	11	14	18
T-2	V-5	45.00	45.00	33.95	29.96	45.00	45.00	45.00	45.00	45.00	45.00	40.33	40.33	45.00	45.00	45.00	45.00	45.00	45.00	45.00	45.00	45.00	45.00	45.00	45.00	45.00	45.00	45.00	45.00	45.00	45.00	45.00
	V-6	45.00	45.00	36.55	38.34	45.00	32.68	45.00	45.00	45.00	45.00	45.00	45.00	45.00	45.00	45.00	45.00	45.00	45.00	45.00	45.00	45.00	45.00	45.00	45.00	38.76	39.22	38.82	45.00	45.00	45.00	45.00
	V-7	45.00	45.00	29.47	34.36	39.69	38.03	45.00	45.00	45.00	45.00	38.51	37.13	45.00	45.00	45.00	45.00	45.00	42.44	45.00	45.00	45.00	45.00	45.00	45.00	45.00	45.00	45.00	45.00	45.00	45.00	45.00
	V-8	45.00	45.00	35.82	36.70	31.86	40.05	37.40	39.69	40.23	36.78	45.00	45.00	45.00	45.00	45.00	45.00	45.00	45.00	45.00	45.00	45.00	45.00	45.00	45.00	45.00	45.00	45.00	45.00	45.00	45.00	45.00
C-2	C-15	45.00	45.00	45.00	45.00	45.00	45.00	45.00	45.00	45.00			No Data				45.00	45.00	45.00	32.36	31.88	D	-	-	-	-	-	-	-	-	-	-
	C-16	45.00	45.00	45.00	45.00	45.00	45.00	45.00	45.00	45.00							45.00	45.00	45.00	28.55	31.17	D	-	-	-	-	-	-	-	-	-	-

Abbreviations: Ct, cycle threshold; DPV, days post-vaccination; DPC, days post-challenge. Values are presented as individual qPCR Ct values for the ASFV p72 gene. According to the assay criteria, samples with Ct values <45 were considered positive, whereas Ct values of 45.000 were considered negative or undetermined at the assay cutoff. "D" indicates that the animal died. "No Data" indicates that the sample was not collected or that the result was unavailable. "-" indicates no available data.

Supplementary Table S9. Individual qPCR Ct values in whole blood samples from the T-3 and C-3 groups.

		Ct (qPCR, Whole Blood)																															
**Group**		**DPV (Days Post-Vaccination)**																										**DPC (Days Post-Challenge)**					
		**0**	**3**	**7**	**10**	**14**	**17**	**21**	**24**	**28**	**31**	**35**	**38**	**42**	**45**	**49**	**52**	**56**	**59**	**63**	**66**	**70**	**73**	**77**	**80**	**84**	**87**	**4**	**7**	**11**	**14**	**18**	**21**
T-3	V-9	45.00	26.08	17.68	21.04	20.56	21.17	22.73	21.43	22.92	23.94	23.61	25.81	23.85	25.62	28.87	27.17	26.61	28.15	28.13	29.52	29.99	29.92	30.44	30.91	30.60	33.20	33.41	33.74	31.68	27.62	29.34	28.74
	V-10	45.00	25.89	17.26	18.24	19.91	20.26	20.12	20.64	21.04	23.11	24.39	25.53	25.16	25.99	28.99	28.74	27.86	28.81	28.84	29.94	29.44	31.02	31.14	31.49	32.36	31.22	35.90	36.80	37.79	37.23	38.86	45.00
	V-11	45.00	45.00	33.33	33.00	19.03	20.42	22.03	22.35	21.12	22.65	23.71	25.39	25.48	27.17	29.79	30.22	29.98	31.12	30.71	31.78	32.46	32.79	33.26	33.58	34.07	34.12	35.58	35.70	38.19	37.44	36.78	38.58
	V-12	45.00	33.69	31.22	33.27	36.34	32.04	33.93	37.46	37.57	37.93	36.86	35.63	45.00	45.00	45.00	45.00	45.00	45.00	45.00	45.00	45.00	45.00	45.00	45.00	45.00	45.00	45.00	45.00	37.81	45.00	45.00	45.00
C-3	C-17	45.00	45.00	45.00	45.00	45.00	45.00	45.00	45.00	45.00								No Data								45.00	45.00	21.78	16.98	17.17	D	-	-
	C-18	45.00	45.00	45.00	45.00	45.00	45.00	45.00	45.00	45.00																45.00	45.00	17.38	16.59	D	-	-	-

Supplementary Table S10. Individual qPCR Ct values in serum samples from the T-3 and C-3 groups.

		Ct (qPCR, Serum)																															
**Group**		**DPV (Days Post-Vaccination)**																										**DPC (Days Post-Challenge)**					
		**0**	**3**	**7**	**10**	**14**	**17**	**21**	**24**	**28**	**31**	**35**	**38**	**42**	**45**	**49**	**52**	**56**	**59**	**63**	**66**	**70**	**73**	**77**	**80**	**84**	**87**	**4**	**7**	**11**	**14**	**18**	**21**
T-3	V-9	45.00	34.56	21.13	23.39	25.55	30.12	28.24	27.73	35.01	36.60	36.14	38.81	37.52	45.00	38.49	36.38	45.00	45.00	45.00	45.00	45.00	45.00	39.77	45.00	45.00	45.00	45.00	45.00	39.85	39.06	38.22	33.43
	V-10	45.00	33.34	23.42	22.48	22.94	27.06	26.59	27.51	34.37	34.17	36.39	37.86	36.13	37.31	45.00	36.53	35.10	45.00	45.00	40.29	45.00	45.00	45.00	45.00	45.00	45.00	45.00	45.00	45.00	45.00	45.00	45.00
	V-11	45.00	45.00	35.10	36.99	22.48	23.44	28.78	33.07	34.87	35.44	36.78	38.21	45.00	45.00	39.17	45.00	45.00	45.00	45.00	45.00	45.00	45.00	45.00	45.00	45.00	45.00	45.00	45.00	45.00	45.00	45.00	45.00
	V-12	45.00	38.93	33.00	45.00	45.00	37.69	39.71	45.00	45.00	45.00	45.00	45.00	45.00	45.00	45.00	45.00	45.00	45.00	45.00	45.00	45.00	45.00	45.00	45.00	45.00	45.00	45.00	45.00	45.00	45.00	45.00	45.00
C-3	C-17	45.00	45.00	45.00	45.00	45.00	45.00	45.00	45.00	45.00								No Data								45.00	45.00	29.70	19.34	19.99	D	-	-
	C-18	45.00	45.00	45.00	45.00	45.00	45.00	45.00	45.00	45.00																45.00	45.00	21.94	17.39	D	-	-	-

Supplementary Table S11. Individual qPCR Ct values in oral swab samples from the T-3 and C-3 groups.

		Ct (qPCR, Oral)																															
**Group**		**DPV (Days Post-Vaccination)**																										**DPC (Days Post-Challenge)**					
		**0**	**3**	**7**	**10**	**14**	**17**	**21**	**24**	**28**	**31**	**35**	**38**	**42**	**45**	**49**	**52**	**56**	**59**	**63**	**66**	**70**	**73**	**77**	**80**	**84**	**87**	**4**	**7**	**11**	**14**	**18**	**21**
T-3	V-9	45.00	45.00	38.88	45.00	40.56	38.94	45.00	38.99	45.00	45.00	45.00	45.00	45.00	45.00	45.00	45.00	45.00	45.00	45.00	45.00	45.00	45.00	45.00	45.00	45.00	45.00	45.00	45.00	45.00	45.00	39.40	38.51
	V-10	45.00	45.00	37.51	40.24	45.00	45.00	45.00	45.00	45.00	45.00	45.00	45.00	45.00	45.00	45.00	45.00	45.00	45.00	45.00	45.00	45.00	45.00	45.00	45.00	45.000	45.00	45.00	45.000	45.00	45.00	45.00	45.00
	V-11	45.00	45.00	37.67	45.00	40.30	41.04	45.00	45.00	45.00	45.00	45.00	45.00	45.00	45.00	45.00	45.00	45.00	45.00	45.00	45.00	45.00	45.00	45.00	45.00	45.00	45.00	45.00	45.00	45.00	45.00	45.00	45.00
	V-12	45.00	45.00	35.61	39.40	39.16	45.00	45.00	45.00	45.00	45.00	45.00	45.00	45.00	45.00	45.00	45.00	45.00	45.00	45.00	45.00	45.00	45.00	45.00	45.00	45.00	45.00	45.00	45.00	45.00	45.00	45.00	38.55
C-3	C-17	45.00	45.00	45.00	45.00	45.00	45.00	45.00	45.00	45.00								No Data								45.00	45.00	34.48	34.09	25.59	D	-	-
	C-18	45.00	45.00	45.00	45.00	45.00	45.00	45.00	45.00	45.00																45.00	45.00	36.04	30.08	D	-	-	-

Supplementary Table S12. Individual qPCR Ct values in rectal samples from the T-3 and C-3 groups.

		Ct (qPCR, Fecal)																															
**Group**		**DPV (Days Post-Vaccination)**																										**DPC (Days Post-Challenge)**					
		**0**	**3**	**7**	**10**	**14**	**17**	**21**	**24**	**28**	**31**	**35**	**38**	**42**	**45**	**49**	**52**	**56**	**59**	**63**	**66**	**70**	**73**	**77**	**80**	**84**	**87**	**4**	**7**	**11**	**14**	**18**	**21**
T-3	V-9	45.00	45.00	32.36	34.44	36.02	36.22	37.24	35.32	33.84	33.66	45.00	45.00	45.00	45.00	45.00	45.00	45.00	39.04	45.00	45.00	45.00	45.00	45.00	45.00	45.00	45.00	45.00	45.00	45.00	38.62	39.35	45.00
	V-10	45.00	45.00	35.58	35.04	45.00	45.00	45.00	38.71	36.05	35.53	45.00	45.00	36.94	45.00	45.00	45.00	45.00	45.00	45.00	45.00	45.00	45.00	45.00	45.00	45.00	45.00	40.35	45.00	45.00	37.98	40.58	40.41
	V-11	45.00	45.00	45.00	45.00	37.81	45.00	45.00	45.00	30.45	45.00	45.00	45.00	45.00	45.00	45.00	45.00	45.00	45.00	45.00	45.00	45.00	45.00	45.00	45.00	45.00	45.00	45.00	45.00	45.00	45.00	45.00	45.00
	V-12	45.00	45.00	45.00	45.00	45.00	45.00	45.00	45.00	45.00	45.00	45.00	45.00	45.00	45.00	45.00	45.00	45.00	45.00	45.00	45.00	45.00	45.00	45.00	45.00	45.00	45.00	45.00	45.00	45.00	45.00	45.00	45.00
C-3	C-17	45.00	45.00	45.00	45.00	45.00	45.00	45.00	45.00	45.00								No Data								45.00	45.00	45.00	31.48	27.18	D	-	-
	C-18	45.00	45.00	45.00	45.00	45.00	45.00	45.00	45.00	45.00																45.00	45.00	36.78	27.29	D	-	-	-

Abbreviations: Ct, cycle threshold; DPV, days post-vaccination; DPC, days post-challenge. Values are presented as individual qPCR Ct values for the ASFV p72 gene. According to the assay criteria, samples with Ct values <45 were considered positive, whereas Ct values of 45.000 were considered negative or undetermined at the assay cutoff. "D" indicates that the animal died. "No Data" indicates that the sample was not collected or that the result was unavailable. "-" indicates no available data.
